# Correlation of an epigenetic mitotic clock with cancer risk

**DOI:** 10.1186/s13059-016-1064-3

**Published:** 2016-10-03

**Authors:** Zhen Yang, Andrew Wong, Diana Kuh, Dirk S. Paul, Vardhman K. Rakyan, R. David Leslie, Shijie C. Zheng, Martin Widschwendter, Stephan Beck, Andrew E. Teschendorff

**Affiliations:** 1 CAS Key Laboratory of Computational Biology, CAS-MPG Partner Institute for Computational Biology, 320 Yue Yang Road, Shanghai, 200031 China; 2 MRC Unit for Lifelong Health and Ageing at University College London, London, UK; 3 Medical Genomics, UCL Cancer Institute, University College London, 72 Huntley Street, London, WC1E 6BT UK; 4 The Blizard Institute, Barts and The London School of Medicine and Dentistry, Queen Mary University of London, London, E1 2AT UK; 5 Department of Women’s Cancer, University College London, 74 Huntley Street, London, WC1E 6AU UK; 6 Statistical Cancer Genomics, Paul O’Gorman Building, UCL Cancer Institute, University College London, 72 Huntley Street, London, WC1E 6BT UK

**Keywords:** DNA methylation, Epigenetic clock, Cancer, Mitotic, Stem cells, Ageing

## Abstract

**Background:**

Variation in cancer risk among somatic tissues has been attributed to variations in the underlying rate of stem cell division. For a given tissue type, variable cancer risk between individuals is thought to be influenced by extrinsic factors which modulate this rate of stem cell division. To date, no molecular mitotic clock has been developed to approximate the number of stem cell divisions in a tissue of an individual and which is correlated with cancer risk.

**Results:**

Here, we integrate mathematical modeling with prior biological knowledge to construct a DNA methylation-based age-correlative model which approximates a mitotic clock in both normal and cancer tissue. By focusing on promoter CpG sites that localize to Polycomb group target genes that are unmethylated in 11 different fetal tissue types, we show that increases in DNA methylation at these sites defines a tick rate which correlates with the estimated rate of stem cell division in normal tissues. Using matched DNA methylation and RNA-seq data, we further show that it correlates with an expression-based mitotic index in cancer tissue. We demonstrate that this mitotic-like clock is universally accelerated in cancer, including pre-cancerous lesions, and that it is also accelerated in normal epithelial cells exposed to a major carcinogen.

**Conclusions:**

Unlike other epigenetic and mutational clocks or the telomere clock, the epigenetic clock proposed here provides a concrete example of a mitotic-like clock which is universally accelerated in cancer and precancerous lesions.

**Electronic supplementary material:**

The online version of this article (doi:10.1186/s13059-016-1064-3) contains supplementary material, which is available to authorized users.

## Background

Estimating the relative rate of stem cell divisions of a given tissue type between individuals may allow their stratification according to their prospective risk of cancer [1, 2]. It is therefore of interest to construct molecular mitotic-like clocks, which may provide an approximate estimate of the relative stem cell division rate of a tissue in an individual [3–5]. While telomere shortening represents a mitotic clock [6] and has been associated with increased cancer risk [7], these associations have, however, been largely inconsistent and only obtained in surrogate tissues such as blood [8]. A recently identified mutational clock-like signature [5] may also approximate a mitotic clock but has not yet been applied to cancer risk prediction.

Errors in the maintenance of DNA methylation (DNAm) arising during cell division may accumulate in the stem cell population of a tissue in line with the stem cell division rate and chronological age and have been proposed as molecular marks for a mitotic clock [3, 4, 9]. In addition, an increased rate of mitosis in the stem cell pool, possibly associated with cancer risk factors such as inflammation or viral infection, has been suggested to fuel epigenetic cellular heterogeneity and to lead to an increased epigenetic clonal mosaicism which may predispose the tissue to future neoplastic transformation [10–15]. Indeed, clonal genetic and copy number variation mosaicism has already been associated with the future risk of hematological cancers [16–19], and DNAm variability in normal cervical cells has been shown to predict the prospective risk of cervical cancer [15]. Given that many cancer risk factors have been associated with DNAm changes in normal cells [12, 15, 20–22], and preferentially at the same sites that undergo DNAm changes with age in healthy tissue [23, 24], we posited that a DNAm based mitotic-like clock could serve as a tool to predict cancer risk.

Here we report substantial progress towards the construction of such an epigenetic mitotic-like clock. Using a novel approach, based on an underlying mathematical model, we build a DNAm-based age-correlative model called “epiTOC” (Epigenetic Timer Of Cancer). A key feature underlying the construction of epiTOC is the focus on Polycomb group target (PCGT) promoter CpGs which are unmethylated in many different fetal tissue types, thus allowing us to define a proper ground state from which to then assess deviations in DNAm in aged tissue. By correlating the tick rate predictions of this model to the rate of stem cell divisions in normal tissue, as well as to an mRNA expression-based mitotic index in cancer tissue, we demonstrate that our model approximates a mitotic-like clock. Importantly, unlike Horvath’s epigenetic clock [25], the tick rate of epiTOC is universally accelerated in cancer, in preinvasive lesions, in normal epithelial cells at risk of neoplastic transformation, and in normal epithelial cells exposed to smoke carcinogens.

## Results

### Construction of the epiTOC model

**Fig. 1:**
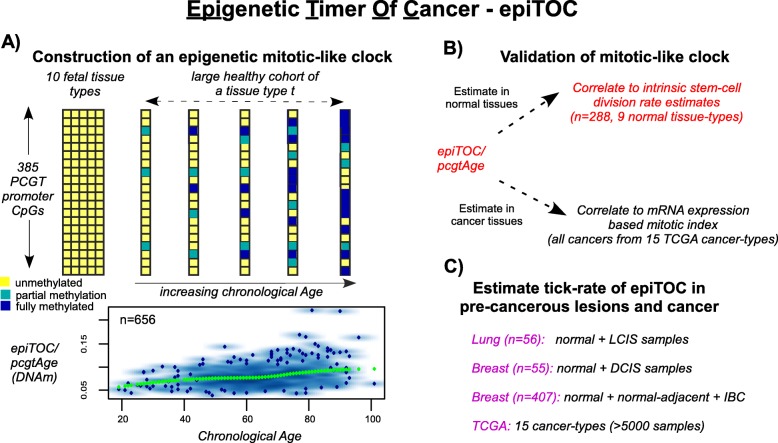
Flowchart of epiTOC. **a** The epiTOC score (also *pcgtAge* score) is estimated over 385 PCGT/PRC2-marked promoter CpGs that are constitutively unmethylated in over 37 fetal tissue samples from 12 tissue types and whose DNAm increases with chronological age in a large cohort of healthy individuals, as assessed in one tissue type (blood). The epiTOC/*pcgtAge* score of any sample represents the average DNAm from the ground fetal state over these 385 sites, representing the life-time accumulation of DNAm replication errors, and provides a relative estimate of the number of stem cell divisions per stem cell. The *lower panel* shows how this score varies linearly with chronological age (data as estimated in the Hannum et al. [28] blood dataset). **b** Validation of the epiTOC/*pcgtAge* score by correlation to the estimated intrinsic rate of stem cell division per stem cell in normal tissue samples from The Cancer Genome Atlas (*TCGA*) for which such estimates are available. Further validation of epiTOC/*pcgtAge* by correlation to an mRNA expression-based mitotic index in cancer tissues from TCGA. **c** Assessment of whether epiTOC/*pcgtAge* is accelerated in precancerous lesions (lung carcinoma in situ (*LCIS*) and ductal carcinoma in situ of the breast (*DCIS*) and cancer (TCGA) and whether it can predict the prospective risk of invasive lung cancer (*ILC*)

By virtue of it being a highly accurate multi-tissue age predictor, Horvath’s clock cannot be a mitotic clock [14, 25]. Thus, in order to construct an age-correlative model which also reflects a mitotic clock-like process, we devised an alternative strategy, integrating mathematical modeling with previous biological knowledge (“Methods”). We reasoned that using only one tissue type from a large cohort of healthy individuals and focusing on CpG sites which, based on previous biological knowledge [26, 27], would more likely capture mitotic effects, relevant CpGs could be identified by correlation with chronological age (“Methods”; Fig. 1a). Specifically, we focused on CpGs satisfying the following criteria (justification in “Methods”): (1) CpGs that are constitutively unmethylated in fetal tissue encompassing many different tissue types [27]; (2) CpGs that map to gene promoters marked by the PRC2 polycomb repressive complex (also known as Polycomb group targets (PCGTs)) in human embryonic stem cells (hESCs) [26]; and (3) CpGs whose DNAm levels increase with chronological age [23]. Briefly, requirement 1 facilitates the construction of a mitotic-like clock since these CpGs all have comparable DNAm levels in a ground state of age zero, ensuring that deviations from this ground state are therefore also comparable between CpGs. Requirements 2 and 3 are justified based on prior biological knowledge that PCGT promoters undergo DNAm increases during hematopoietic ontogeny [26] and that they define age-associated signatures which are valid across different normal tissue types [23], including purified blood [24] and stem cell populations [23].

Using one of the largest Illumina 450 k DNAm datasets encompassing over 650 whole blood samples from healthy individuals spanning an age range of over 80 years [28] and correcting for changes in blood cell subtype composition (“Methods”; Additional file 1), we identified a subset of 385 PCGT promoter CpGs satisfying all required properties, including being unmethylated across 11 different fetal tissue types and exhibiting age-associated hypermethylation (false discovery rate <0.05) (“Methods”; Additional file 2). For each sample, epiTOC yields a score, denoted “*pcgtAge*”, as the average DNAm over these 385 CpG sites, representing the age-cumulative increase in DNAm at these sites due to putative cell-replication errors (Figure S1a in Additional file 3). Given that hypomethylation is also commonly observed in aging and cancer [29, 30], a separate age-correlative model based on promoter CpGs that are partially methylated in fetal tissue and which become hypomethylated with age was also derived (“Methods”; Figure S1b in Additional file 3). We validated both age-correlative models in an independent Illumina 450 k data set, encompassing over 300 whole blood samples from healthy individuals [31] (Figure S2 in Additional file 3). However, only epiTOC correlated with age in other normal tissue types (Figure S3 in Additional file 3), with the age-associated hypomethylation model showing inconsistent patterns (Figure S4 in Additional file 3).

### epiTOC correlates with age in purified cell and stem cell populations

**Fig. 2:**
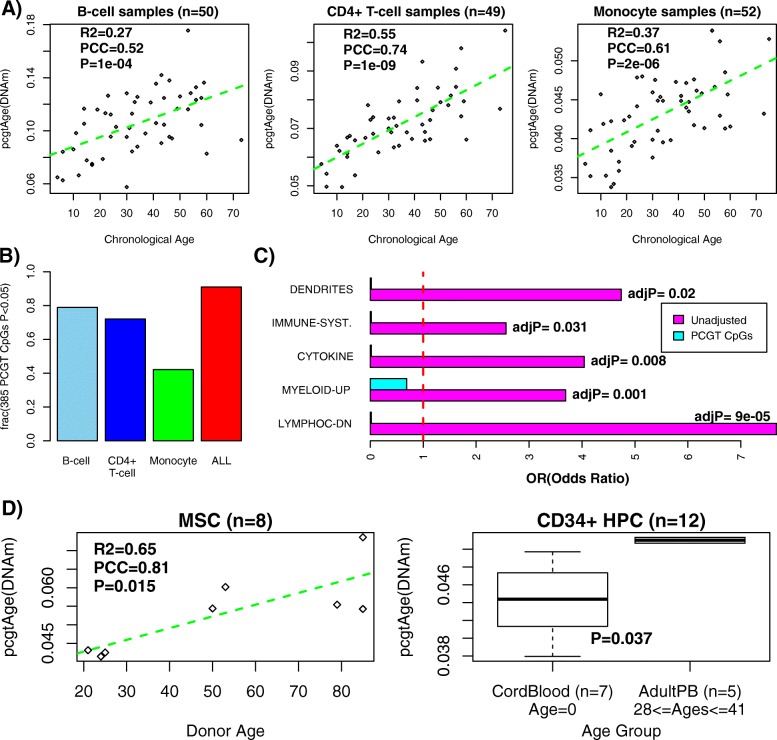
epiTOC correlates with chronological age in purified cell and stem cell populations and is independent of blood cell type composition. **a** *pcgtAge* versus chronological age in three purified blood cell subtype sample sets, as indicated. Number of samples is indicated at the top of each panel. The *green dashed line* is a linear least squares fit. R^2^ value, Pearson correlation coefficient (*PCC*) and linear regression *P* values are given. **b** The fraction of the 385 PCGT CpGs that make up epiTOC which are significantly hypermethylated with age in each of these three purified sample sets, as well as the fraction of these 385 CpGs which are significantly hypermethylated with age in at least one of these three sets (*ALL*, *red*). **c** Gene set enrichment analysis odds ratios (*OR*) of immune and blood cell subtype terms that are highly enriched among age-associated promoter CpGs in Hannum et al. [28] without adjustment for cellular heterogeneity (*Unadjusted*, *magenta bars*). Adjusted *P* values (*adjP*) are given. ORs for these same biological terms among the 385 PCGT CpGs of epiTOC are also shown (*cyan bars*), indicating strong underenrichment. **d** *Left panel*: Correlation of the *pcgtAge* score with donor age of eight bone marrow-derived mesenchymal stem cell (*MSC*) populations (all of low passage). R^2^ value, Pearson correlation coefficient (*PCC*) and linear regression *P* value are given. *Right panel:* Correlation of *pcgtAge* score with donor age of 12 CD34+ hematopoietic progenitor cell (*HPC*) populations. Here we used a Wilcoxon rank sum test to derive a *P* value since there are two well defined groups of cord blood (n = 7, age zero) and adult peripheral blood (*AdultPB*, n = 5) samples

Although epiTOC was constructed by correcting for cellular heterogeneity in blood, age-associated DNAm changes could be non-linear and therefore linear correction for cell type composition may not effectively remove the effect of this confounder [32]. Hence, we sought to reconfirm that the identification of our 385 PCGT promoter CpGs was not affected by age-associated changes in blood cell type composition. To this end, we analyzed Illumina 450 k data from a cohort of healthy individuals spanning a wide age range of over 70 years, obtained from purified cells sorted using FACS and representing three different blood cell subtypes (CD4+ T cells, CD14+/CD16− monocytes, and CD19+ B cells) and encompassing 151 independent samples (“Methods”). In all three cell subtypes, the *pcgtAge* score correlated very significantly with chronological age (Fig. 2a; linear regression *P* = 0.0001 for B cells, *P* = 1e-9 for CD4+ T cells, and *P* = 2e-6 for monocytes). Despite the relatively small sample size of each purified sample set (n ~ 50), a relatively large fraction of the 385 PCGT CpGs were significantly hypermethylated with age in each set, with 91 % of the 385 PCGT CpGs correlating with age in at least one of these purified sample subsets (Fig. 2b). Further attesting that epiTOC correlates with chronological age independently of changes in cell type composition, we observed that *pcgtAge* also increased significantly with age in two additional purified cell 450 k sets profiling a larger set of samples (214 CD4+ T cell and 1202 monocyte samples) but spanning a much lower age range of ~40 years [33] (linear regression *P* < 1e-5 for T cells and *P* < 1e-9 for monocytes) (Figure S5 in Additional file 3).

We also performed a gene set enrichment analysis on the 385 PCGT CpGs that make up epiTOC to see if there is any evidence for these CpGs mapping to immune/blood cell subtype markers. To identify relevant blood cell or immune cell type terms, we first conducted the gene set enrichment analysis on top ranked CpGs in the Hannum et al. [28] data without correction for cellular heterogeneity, which, as expected, revealed strong enrichment of promoter CpGs mapping to genes underexpressed in lymphocytes and genes overexpressed in myeloid cells (Fig. 2c), consistent with the known increased myeloid–lymphoid ratio with age [34]. In contrast, these same biological terms were conspicuously absent and underenriched among the 385 PCGT epiTOC CpGs (Fig. 2c; Additional file 4).

Finally, we also assessed epiTOC in stem cell populations in order to support our underlying assumption that DNAm alterations at the epiTOC PCGT loci can accrue with age in a stem cell pool. We obtained Illumina Infinium 27 k DNA methylation data for a total of eight bone marrow-derived mesenchymal stem cell (MSC) populations of low passage number, representing a wide donor age range (20–80 years) [35], as well as for 12 CD34+ hematopoietic progenitor cell (HPC) populations derived from cord blood and adult peripheral blood [36]. In both studies, and despite the small sample sizes, the *pcgtAge* score correlated positively with donor age (linear regression *P* = 0.015 for MSCs and Wilcoxon rank sum test *P* = 0.037 for HPCs; Fig. 2d).

### epiTOC approximates a mitotic clock

**Fig. 3:**
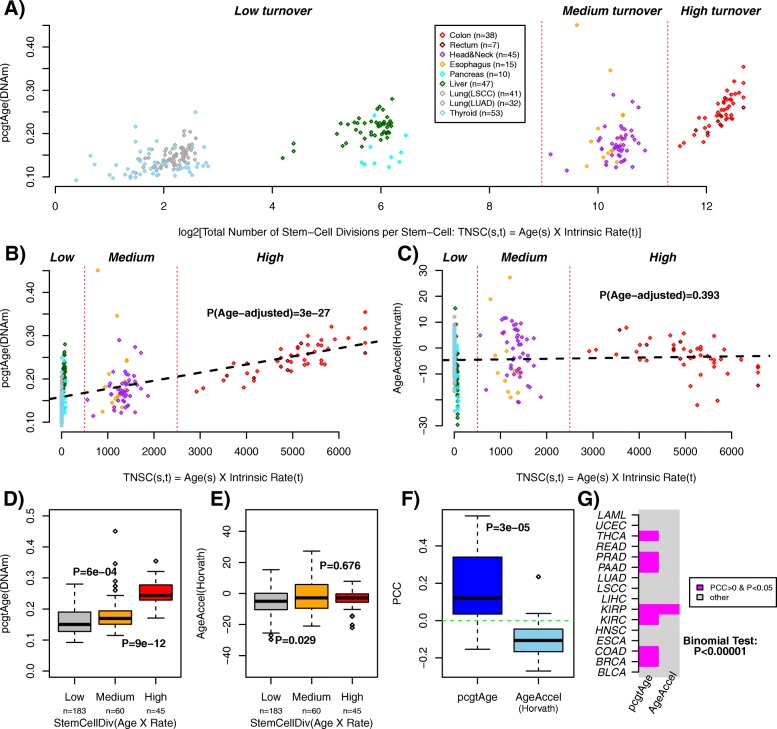
The *pcgtAge*/epiTOC model approximates a mitotic clock. **a** The *x-axis* labels the cumulative total number of stem cell divisions (TNSC) incurred per stem cell in the given normal tissue in a log_2_ scale. This number is the log_2_ of the product of the intrinsic rate of cell divisions per year per stem cell (tissue-dependent) with the chronological age of the sample (tissue-independent). The *y-axis* labels the *pcgtAge* score of the sample. Samples have been colored according to the normal tissue of origin (normal samples adjacent to TCGA cancer types, as indicated). The number of normal samples for each tissue is given. Samples are seen to cluster into three main groups: a low cellular turnover group, a medium cellular turnover group, and a high cellular turnover group (note that this grouping is inferred from the unlogged scale, as shown in **b**). **b** Same as panel **a** but with TNSC shown in the unlogged scale. The *P* value is from a linear regression adjusted for chronological age of samples. **c** As **b** but for Horvath’s age-acceleration measure (i.e., DNAmAge minus chronological age). The *P* value is from a linear regression (already adjusted for age from the definition of age acceleration). **d** *pcgtAge* scores for the three different turnover groups as shown in **a** and **b**. The *P* values are from comparing the neighboring groups using a one-tailed Wilcoxon rank sum test, adjusted for chronological age. **e** As **d** but for Horvath’s age-acceleration measure. **f** The Pearson correlation coefficient (*PCC*) between either *pcgtAge* or Horvath’s age-acceleration measure, with an mRNA expression-based mitotic index, as estimated over all cancer samples of a given cancer type for a total of 15 different TCGA cancer types. Thus, each boxplot has 15 data points. The *P* value is from a one-tailed paired Wilcoxon rank sum test. **g** Corresponding heat map indicating for which cancer types there was a significant positive PCC between *pcgtAge* or Horvath’s AgeAccel with the mRNA expression-based mitotic index. The binomial test *P* value indicates that *pcgtAge* correlates more strongly with the mitotic index than Horvath’s age-acceleration measure

To demonstrate that epiTOC approximates a mitotic-like clock we computed the *pcgtAge* score in 288 normal samples from nine different tissue types collected from TCGA consortium [37] and for which independent estimates of the intrinsic stem cell division rates were available [2, 38] (Fig. 1b). Using the chronological age of the sample and the intrinsic cell division rate of the tissue, we obtained estimates of the cumulative total number of divisions incurred per stem cell in each sample (TNSC). Plotting these TNSC estimates on a log scale showed that samples spread mainly according to tissue type and secondly by age (Fig. 3a). On the natural unlogged scale, it revealed that the 288 normal samples clustered into three groups, characterized by a low, intermediate, and high cellular turnover (Fig. 3b). Fitting a linear regression, adjusted for chronological age, between the predicted *pcgtAge* from our model and the total number of stem cell divisions per stem cell in the sample revealed a strong positive correlation (*P* < 1e-26, R^2^ = 0.45; Fig. 3b). Differences in *pcgtAge* between the cellular turnover groups were also statistically significant (Fig. 3d). As a negative control, the corresponding correlation between Horvath’s measure of age acceleration [25] and TNSC was either not significant (P = 0.39, R^2^ ~ 0; Fig. 3c) or, in the case of between group comparisons, of only marginal significance (Fig. 3e).

To further demonstrate that *pcgtAge* provides a correlative measure of the cell division rate in a tissue, we posited that it would correlate with an mRNA expression-based mitotic index, which we constructed from the expression levels of genes that have been highly validated as being cell proliferation markers (“Methods”). This expression-based mitotic index was increased in all 15 TCGA cancer types compared to their corresponding normal tissue (Figures S6 and S7 in Additional file 3) and also correlated strongly with the TNSC estimates across normal tissues (*P* < 1e-21; Figure S8 in Additional file 3). Focusing on only the cancer samples from each of these cancer types, we obtained Pearson correlations between their *pcgtAge* and their expression-based mitotic index. Correlations were generally positive and much higher than those obtained using Horvath’s age-acceleration measure (Fig. 3f; paired Wilcoxon rank sum test *P* < 0.0001). For *pcgtAge* we observed a significant (*P* < 0.05) positive correlation in 7/15 cancer types, whilst for Horvath’s clock only one cancer type (KIRP) exhibited such an association (Fig. 3g). All these data support the view that epiTOC represents an approximate mitotic-like clock. In contrast, the model based on age-associated hypomethylation did not correlate well with the expression-based mitotic index in cancer tissue, although we did observe an excellent correlation with cellular turnover rates in normal tissue (Figure S9 in Additional file 3).

### epiTOC predicts universal age acceleration in cancer and is further increased in cancer cell lines

**Fig. 4:**
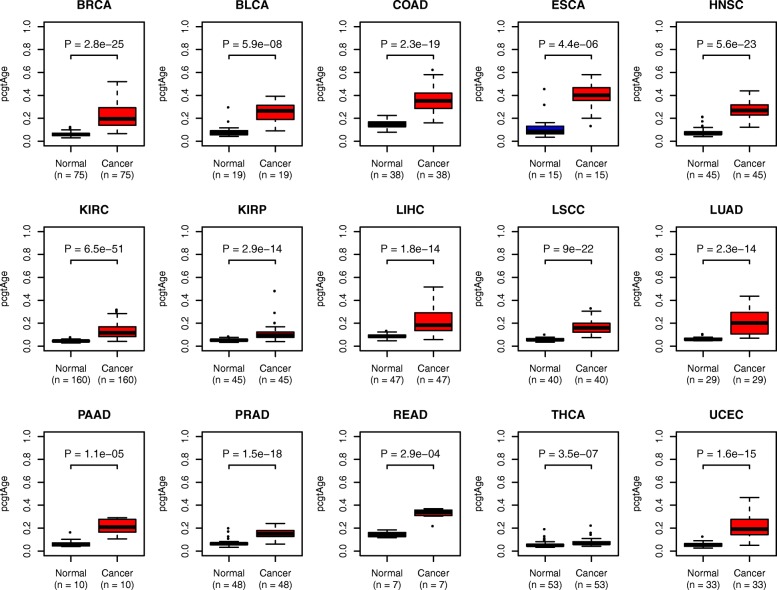
The *pcgtAge*/epiTOC model predicts universal age acceleration in cancer. Boxplots comparing the *pcgtAge* of age-matched normal cancer samples across 15 TCGA cancer types. *P* values are from a paired (one-tailed) Wilcoxon rank sum test. *BRCA* breast adenocarcinoma; *BLCA* bladder adenocarcinoma; *COAD* colon adenocarcinoma; *ESCA* esophageal cancer; *HNSC* head & neck squamous cell carcinoma; *KIRC* kidney renal cell carcinoma; *KIRP* kidney renal papillary carcinoma; *LIHC* liver hepatocellular carcinoma; *LSCC* lung squamous cell carcinoma; *LUAD* lung adenocarcinoma; *PAAD* pancreatic adenocarcinoma; *PRAD* prostate adenocarcinoma; *READ* rectal adenocarcinoma; *THCA* thyroid carcinoma; *UCEC* uterine corpus endometrial carcinoma

Because cell proliferation is a cancer hallmark, we reasoned that epiTOC would predict an accelerated tick rate in all cancer types (Fig. 1c). We confirmed this using all age-matched normal–tumor pairs from 15 TCGA cancer types (Fig. 4). In contrast, Horvath’s clock and the age-hypomethylated CpG-based model did not consistently predict age acceleration in cancer (Figures S10 and S11 in Additional file 3). Of note, the *pcgtAge* score also outperformed the mRNA-based mitotic index, as a discriminator of normal/cancer status, in 9/15 cancer types (Figure S12 in Additional file 3).

Next, we asked if the *pcgtAge* score is also increased in cancer cell lines compared to cell lines of normal karyotype. Using Illumina 450 k DNAm data from 24 cancer and 29 normal cell lines, all profiled as part of ENCODE [39], we observed a significantly higher *pcgtAge* score in the cancer cell lines (Wilcoxon rank sum test *P* = 6e-14; Figure S13a in Additional file 3). Cancer cell lines generally also exhibited higher *pcgtAge* scores than those observed in cancer tissue. To confirm this, we compared the *pcgtAge* scores for 11 cancer types against the *pcgtAge* score of a corresponding representative cancer cell line (“Methods”). In all 11 cancer types, the cancer cell line exhibited a higher score than the average over corresponding cancer tissue samples (Wilcoxon paired rank sum test *P* = 0.005; Figure S13b in Additional file 3).

### Increased epiTOC tick rate in pre-invasive cancer lesions

**Fig. 5:**
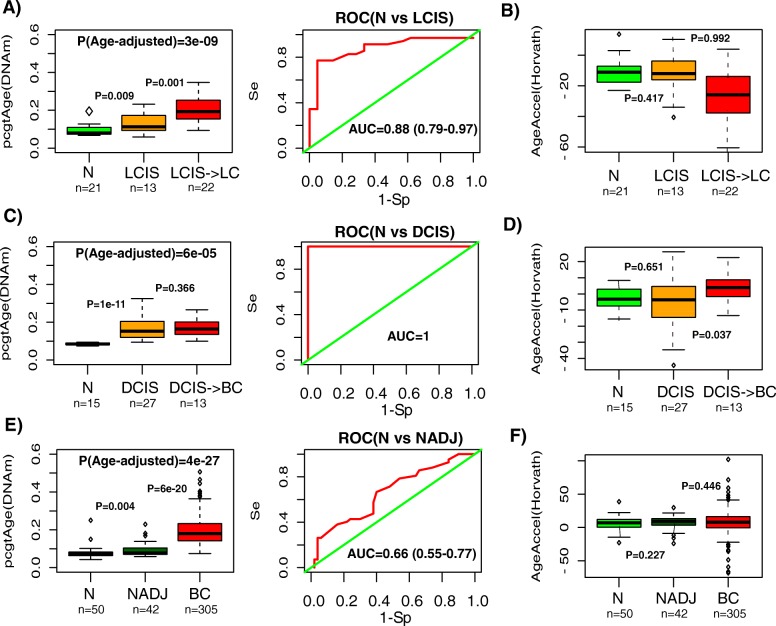
*pcgtAge*/epiTOC is aggravated in pre-cancerous lesions whereas Horvath’s age-acceleration measure is not. **a** *Left panel*: the *pcgtAge* between normal lung tissue (*N*), lung carcinoma in situ (*LCIS*), and LCIS samples from patients who later progressed to an invasive lung cancer (*LCIS- > LC*). *P* values between respective groups are from a one-tailed Wilcoxon rank sum test. *P* value from a linear regression of *pcgtAge* versus group adjusted for age is also given at the top. *Right panel*: corresponding receiver operating characteristic (*ROC*) for discriminating the normal and all LCIS samples. AUC and 95 % CI is given. **b** Horvath’s age-acceleration measure between normal lung tissue (*N*), lung carcinoma in situ (*LCIS*), and LCIS samples from patients who later progressed to an invasive lung cancer (*LCIS- > LC*). *P* values between respective groups are from a one-tailed Wilcoxon rank sum test. **c**, **d** Exactly as **a**, **b** but now in a different Illumina 450 k data set, encompassing normal breast tissue, ductal carcinoma in situ (*DCIS*), and DCIS samples from women who later developed an invasive breast cancer (*BC*). **e**, **f** Exactly as **a**, **b** but now in an Illumina 450 k set which profiled normal breast tissue from healthy women (*N*), normal breast tissue adjacent to a breast cancer (*NADJ*), and breast cancers (*BC*). The number of samples in each group is given below

We further reasoned that *pcgtAge* might also be increased in pre-cancerous tissue owing to a marginal increase in cell proliferation. We computed *pcgtAge* in an independent Illumina 450 k dataset encompassing 21 normal lung tissue samples and 35 lung carcinoma in situ (LCIS) samples, of which 22 progressed to an invasive lung cancer [20]. This revealed a gradual increase in *pcgtAge* from normal lung, to LCIS, and to LCIS which progressed to invasive lung cancer (ILC), a result which was independent of chronological age (Fig. 5a). *pcgtAge* could discriminate normal lung from LCIS with an AUC of 0.88 (95 % confidence interval (CI) 0.79–0.97; Fig. 5a), as well as discriminating the LCIS which progressed to ILC from those which did not (AUC = 0.79, 95 % CI 0.63–0.94; Figure S14a in Additional file 3). In contrast to epiTOC, Horvath’s clock did not predict age acceleration in the LCIS samples and LCIS samples which progressed to ILC even exhibited substantial age deceleration (Fig. 5b). The age-hypomethylated model could neither discriminate normal from LCIS (Figure S15 in Additional file 3) nor predict cancer risk (Figure S14b in Additional file 3).

Most of these results were replicated in an Illumina 450 k dataset encompassing 14 normal breast tissues (from reduction surgery), 28 age-matched ductal carcinomas in situ (DCIS), and a further 13 age-matched DCIS samples from women who later developed an invasive breast cancer (IBC) [40] (Fig. 5c). Specifically, we observed an increased *pcgtAge* score in DCIS compared to normal breast (AUC = 1, *P* < 0.05; Fig. 5c). In contrast, neither Horvath’s measure of age acceleration nor the age-associated hypomethylation model could discriminate normal breast from DCIS samples (Fig. 5d; Figure S16 in Additional file 3). In another independent Illumina 450 k set which profiled 50 normal breast samples, 42 age-matched normal samples adjacent to a breast cancer and an additional 305 breast cancers [41], *pcgtAge* also showed a gradual age-independent increase between normal breast tissue and normal-adjacent breast tissue and breast cancer and was able to discriminate normal breast from normal-adjacent tissue (AUC = 0.66, 95 % CI 0.55–0.77; Fig. 5e). Again, this was not the case for Horvath’s age-acceleration measure nor for the analogous age-associated hypomethylation signature (Fig. 5f; Figure S17 in Additional file 3).

To translate the discrimination accuracies above into an estimate of the difference in incurred stem cell divisions, we integrated the estimate of the intrinsic rate of stem cell division in lung tissue [2, 38] with the epiTOC model, using the normal lung samples from TCGA to estimate the intercept and slope in our regression model (“Methods”). We obtained an estimate of 8.45 stem cell divisions per stem cell in the normal tissue compared to 13.33 divisions per stem cell in LCIS which did not progress to ILC and 22.02 divisions in the LCIS samples which progressed to ILC, representing an approximate threefold increase compared to normal samples. In the case of breast tissue, the underlying rate of stem cell division is unknown, but we could nevertheless estimate that DCIS samples had a 3.5-fold higher number of total stem cell divisions per stem cell compared to age-matched normal breast, a ratio similar to that of LCIS to normal lung tissue.

### Increased epiTOC tickrate in normal buccal tissue of smokers

**Fig. 6:**
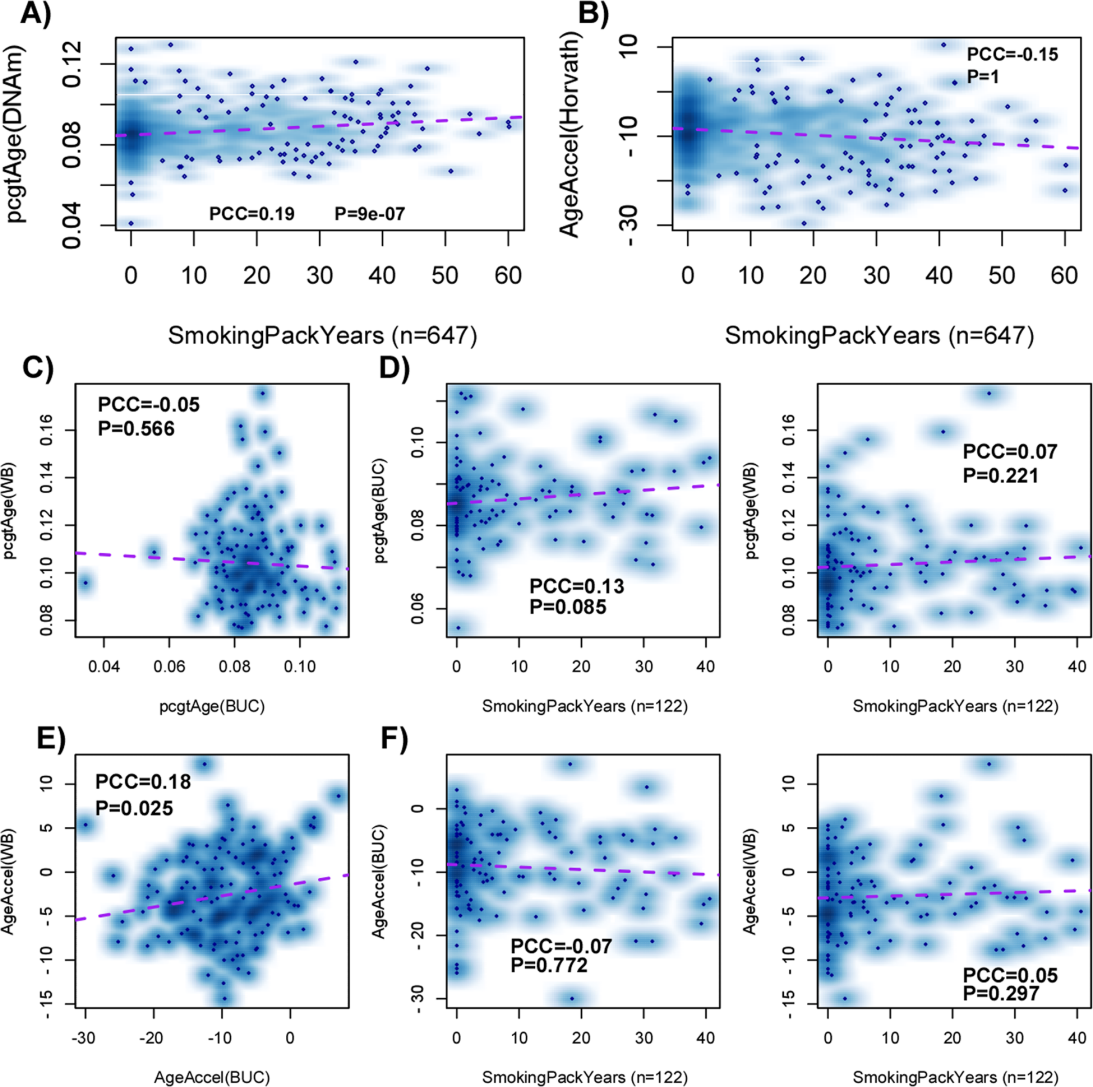
epiTOC’s tick rate is increased in buccal tissue from smokers. **a** Density scatterplot of *pcgtAge* (*y-axis*) versus smoking pack-years for a total of 647 buccal samples from women all aged 53 years at the time of sampling. Pearson correlation coefficient (*PCC*) and one-tailed *P* value from a linear regression are given. **b** As **a** but for Horvath’s age-acceleration measure. **c** Density scatterplot of *pcgtAge* (*y-axis*) in whole blood (WB) versus *pcgtAge* in buccal (*BUC*) tissue for a total of 152 women all aged 53 years who provided both a WB and a buccal sample. PCC and associated one-tailed *P* value are given. **d** Density scatterplots of *pcgtAge* in WB and BUC tissue against smoking pack years for 122 women with smoking pack year information. *P* values are from a one-tailed correlation test. **e**, **f** As **c**, **d** but for Horvath’s age acceleration measure. Note that because all samples are of the same age, the age-acceleration measure is just a constant shift of the predicted DNAm age

Next, we asked if epiTOC’s tick rate is increased in normal cells exposed to a major carcinogen. Given that smoking is a well-established major cancer risk factor for epithelial cancers like lung cancer [42, 43] and that smoking has been shown to be strongly associated with DNAm changes in epithelial cells, specifically in buccal tissue [20], we hypothesized that epiTOC would exhibit an accelerated rate in the buccal tissue of smokers compared to non-smokers. We estimated *pcgtAge* in a large cohort of 790 buccal samples from women all aged 53 years at the time of sampling and for which DNAm data with Illumina 450 k arrays had been generated and smoking pack-year (SPY) information was available for 647 of these women [20]. We focused on SPY as opposed to smoking status at the time of sampling given that SPY better reflects the smoking history of women [20]. Consistent with a model in which smoke carcinogens cause inflammation [44] and in turn an increased mitotic rate [12], the *pcgtAge* score significantly correlated with SPY (linear regression *P* < 1e-6; Fig. 6a). However, substantial variation in the *pcgtAge* scores unrelated to smoking exposure was also evident (Fig. 6a). In contrast, Horvath’s measure of age acceleration did not correlate with SPY (Fig. 6b), although the age-hypomethylated model did show a strong association (Figure S18 in Additional file 3).

For a subset of 152 women for which there was matched blood–buccal tissue, we found that the *pcgtAge* score in the two tissues did not correlate with each other (Fig. 6c), consistent with the fact that all women were of the same age and that the effect of inflammation (and hence cancer risk) is tissue-specific. In line with this, the association of the *pcgtAge* score with SPY was stronger in buccal tissue than in blood (Fig. 6d). In contrast, for Horvath’s age-acceleration measure, which in this cohort of identically aged women equals the predicted DNAm age, we observed a weak yet significant correlation across the two different tissues (Fig. 6e). Correspondingly, no association between Horvath’s age-acceleration measure and SPY was observed, irrespective of tissue type (Fig. 6f).

## Discussion

Unlike telomere shortening [6–8], or the recently identified mutational clock-like signature [5], epiTOC provides a concrete example of a molecular mitotic-like clock which predicts universal acceleration in cancer. Although we acknowledge that current estimates of stem cell division rates in normal tissues are only very rough approximations, the observed correlation between epiTOC and the number of cell divisions per stem cell in over 288 normal samples was obtained by broadly categorizing tissues into groups of low, medium, and high cellular turnover, a classification which is likely to be robust (i.e., it is well accepted that colon has one of the highest cellular turnover rates of any tissue). Importantly, the *pcgtAge* score from epiTOC further correlated with an expression-based mitotic index in as many as seven different cancer types. In those cancer types where we did not see an association with the expression-based mitotic index, this could be due to small differences in proliferation rates between the actual tumors. Supporting this, the *pcgtAge* score was universally increased in cancer, as assessed in 15 tumor types encompassing over 5000 samples, in line with an increased mitotic rate being a universal cancer hallmark. The *pcgtAge* score was also accelerated in preinvasive cancer lesions, including LCIS and breast DCIS samples, allowing highly accurate discrimination of such lesions (AUC ~ 0.9–1). In addition, epiTOC was able to predict risk of an ILC, providing highly accurate identification (AUC ~ 0.8) of LCIS which progressed to an ILC (Figure S14a in Additional file 3). The estimated epiTOC tick rate in normal breast tissue adjacent to breast cancer was also accelerated, albeit at a much lower level, consistent with most of the cells in these samples being of normal cytology. Of note, we recently demonstrated the existence of DNAm field defects in the same normal-adjacent breast tissue samples [41]. That the *pcgtAge* score could discriminate normal tissue containing such field defects from the normal tissue of age-matched cancer-free women (AUC of 0.66, 95 % CI 0.55–0.77) suggests that epiTOC may serve to assess the risk of neoplastic transformation of normal tissue. Large prospective studies will, however, be required to demonstrate utility of epiTOC in a clinical setting.

Of note, the *pcgtAge* score also correlated with SPY in normal buccal tissue, highlighting the potential of epiTOC to capture putative increased cellular proliferation of epithelial cells that have been exposed to a major carcinogen. Although we did not measure inflammatory or cell proliferation markers in our buccal samples, previous studies have noted increased inflammation and proliferative activity in the oral epithelium of smokers [44, 45]. Although it is also plausible that the DNAm changes seen in the buccal epithelium of smokers reflects active changes associated with smoking-induced DNA damage, there is another observation supporting the view that inflammation and increased proliferation underlie most of the observed changes: smoking-associated DNAm changes seen in the buccal epithelium are generally very similar to those seen in healthy aging [20, 46]—for instance, as seen in blood cells from pediatric populations [47, 48]—or similar to those DNAm changes seen in inflammatory diseases [12, 49]. Indeed, a recent review highlighted enrichment of PCGT CpGs as a common DNAm signature which is seen in healthy aging of normal cells (which are presumably free of DNA damage), as well as in normal cells of the same chronological age but which have been exposed to a variety of different cancer risk factors [14]. Hence, the more likely explanation for the observed increased *pcgtAge* score in the buccal epithelial cells of smokers compared to non-smokers of the same age is a marginally increased proliferative activity, possibly associated with an inflammatory response to smoke toxins.

Assessment of epiTOC in cancer cell lines was less conclusive. Although cancer cell lines always exhibited a higher *pcgtAge* score than the average value of the corresponding cancer tissue, consistent with our epiTOC model and with reports that cancer cell lines exhibit higher levels of promoter methylation than cancer tissue [50], comparisons between cell lines and cancer tissue is difficult for a number of reasons. First, studies have shown that the artificial microenvironment and selection pressures of cell culture conditions can modify the genomic, DNAm, and expression patterns of the parental cells [35, 51–53]. Therefore, it is unclear whether the mechanism underlying DNAm changes upon cell division in tumors is also operative in cell lines. Second, a cell line derives from a parent cell which may grossly underrepresent the level of epigenetic heterogeneity in the stem cell pool of the primary cancer tissue, rendering comparisons between cell lines and independent cancer tissue samples problematic. Third, many cell lines would be needed to render meaningful comparisons with cancer tissue [54]. Notwithstanding these limitations, our data are broadly supportive of epiTOC, assuming that, once cells are cultured in vitro, the *pcgtAge* score ceases to reflect the mitotic age of the cells while still reflecting the mitotic age of the parent tissue.

The data presented here further confirm that Horvath’s clock is not a mitotic clock and that it does not exhibit a consistent universal acceleration in cancer or precancerous lesions. Indeed, by its very nature, Horvath’s clock was designed to predict chronological age independently of tissue type and must, therefore, reflect a biological process unrelated to cell division (since cell division rate is highly variable between tissues). While it is not yet clear what this biological process may be, the data presented here indicate that it is not a process that is uniformly altered in cancer, nor a process which appears accelerated in response to a major cancer risk factor such as smoking. In comparing epiTOC to Horvath’s clock, it is also important to point out that epiTOC does not attempt to predict chronological age of samples in absolute terms. This is not a limitation but, in fact, a key advantage of our approach, since any optimization procedure aimed at predicting chronological age as accurately as possible would not allow us to reliably identify the CpGs which reflect an underlying mitotic process.

Importantly, our work has further exposed a subtle difference between promoter CpGs that undergo age-associated hypermethylation from those that undergo hypomethylation, with the analogous model based on age-hypomethylated sites not correlating with the mitotic index in cancer tissue and correspondingly not exhibiting a consistent acceleration in cancer. This is consistent with a recent report by Lin and Wagner [55]. In fact, whereas the model based on hypomethylation also correlated with the number of stem cell divisions per stem cell in normal tissues, as well as with exposure to smoking in normal buccal tissue, it is intriguing that no consistent associations were found in cancer or preinvasive cancer lesions. This suggests that DNA hypermethylation errors associated with cell replication might be similar in cancer and normal stem cells but not so for the corresponding hypomethylation aberrations. It will be important for future studies to try to understand this deep and subtle asymmetry between age-associated hyper- and hypomethylation in relation to the changes seen in cancer.

Interestingly, the mRNA expression-based mitotic index also correlated with the estimated number of stem cell divisions, with the strength of correlation comparable to that obtained by epiTOC. Both mitotic indices were also comparable predictors of normal/cancer status (Figure S12 in Additional file 3). However, only the DNAm-based mitotic index correlates with chronological age. Indeed, it has been shown that mRNA expression signatures correlating with age are not strongly consistent or reproducible across independent studies and that the only consistent genes map to immune system and metabolic pathways and not to cell proliferation [56]. Thus, epiTOC has a higher sensitivity to detect differences in cell division numbers between differently aged samples of the same tissue type. Moreover, epiTOC could discriminate normal breast tissue adjacent to breast cancer from the corresponding normal tissue of age-matched healthy women (Fig. 5e). Although matched mRNA data were not available to assess this for an expression-based mitotic index, it is unclear whether expression based assays can offer the stability and reliability of a DNA-based assay to detect such subtle differences [57].

Although epiTOC correlates with stem cell division rate estimates in normal tissues and correctly predicts an increased tick rate in precancerous and cancer lesions, it is important to point out several limitations underlying our model. First, epiTOC assumes that the underlying DNAm errors are occurring in the underlying stem cell pool of a tissue and ignores active DNAm changes which may occur post-mitotically in response to exposure to various risk factors. As such, the epiTOC model may only capture one part of a more general cancer risk-predictive DNAm signature. However, by computing the *pcgtAge* score in populations of mesenchymal stem cells and hematopoietic progenitor cells, we have seen that epiTOC does predict an increased mitotic age in older stem/progenitor cell populations, supporting the view that it may measure mitotic age of the stem cell pool in epithelial tissues. A second limitation of our model is that it largely ignores absolute stem cell numbers in the tissue of interest, which may vary substantially between individuals and even within an individual as a function of current exposure to environmental factors. The absolute number of stem cells in a tissue is expected to be an important determinant of epigenetic stem cell heterogeneity and clonal mosaicism and, therefore, of cancer risk, as proposed by a number of studies [10, 13, 58]. This is therefore an important caveat, since our model does not distinguish between a tissue with a lower number of stem cells carrying lots of DNAm changes from another tissue with a large number of stem cells, each carrying only a few yet unique DNAm alterations. Nevertheless, assuming similar stem cell numbers, our *pcgtAge* score would be expected to increase in line with the level of epigenetic heterogeneity in the stem cell pool and thus be informative of cancer risk. It is also worth stressing that the observed correlation of the *pcgtAge* score with the tissue’s cellular turnover rate does not depend on any estimates of the actual number of stem cells in the tissue of interest. Indeed, the *pcgtAge* score is an intensive variable, defined as an average over cells and a specific set of loci, and, as such, correlation can only be assessed meaningfully relative to another intensive variable. This other intensive variable is the cumulative number of cell divisions per stem cell in the tissue of interest and not the extensive variable defined as the total number of stem cell divisions in the tissue. For this reason, our model does not make, and cannot make, any predictions of cancer risk between different tissues.

In spite of these limitations, epiTOC may allow, in principle, prediction of stem cell division numbers if absolute stem cell numbers remain constant, if the intrinsic division rate in a tissue is known and if a large DNAm data set of that tissue from healthy individuals is available. In the case of lung tissue, for which estimates of the intrinsic rate of stem cell division are available [2, 38], and using normal-adjacent samples from the TCGA to train the model, we found that LCIS samples exhibited a total of about 13 to 22 divisions per stem cell. This is significantly larger than the approximate eight to nine divisions per stem cell for the normal lung samples, which in turn is only marginally higher than the life-time number of divisions per stem cell in lung tissue (approximately six divisions per stem cell) as estimated by Tomasetti and Vogelstein [2]. Given that it has been estimated that lung tissue contains about one billion stem cells, and assuming that a 2 cm sized LCIS contains 0.5 % of these stem cells, this translates into approximately (22/8) × 0.005 × 10^9^ ~ 14 million additional cell divisions in LCIS compared to a normal sample. We acknowledge that these estimates are only very rough approximations, yet they are also not necessary for the purpose of cancer risk prediction where only the tick rate relative to that of a normal reference tissue is required.

Finally, it is of interest to discuss the relation of epiTOC to a recently proposed “Big Bang” model of early cancer evolution as proposed by Sottoriva et al. [59]. This Big Bang model describes the clonal expansion of a tumor and can accurately explain the observed patterns of spatial genetic heterogeneity within the tumor. In contrast, epiTOC provides an estimate of the relative number of stem cell divisions per stem cell in a tissue of interest, which may approximate the level of epigenetic stem cell heterogeneity within the normal tissue *before* the cancer clone emerges. Once the cancer clone has emerged, the increased cellular proliferation of this clone would be expected to generate a “large wave” of further DNAm changes that may affect most of the genome. Thus, when applying epiTOC to cancer tissue, the *pcgtAge* score is expected to correlate strongly with the level of proliferation of the tumor, and we have indeed demonstrated this in as many as 15 TCGA cancer types. Moreover, we recently demonstrated that the pattern of aberrant DNAm of one tumor sample can explain, on average, about 60 % (R^2^ ~ 0.6) of the aberrant DNAm variation of another tumor, even if from a different cancer type [60], suggesting that a common process underlies most of the DNAm variation in the cancer genome. This common universal process is likely to be an increased cell division rate. The epigenetic epiTOC and genetic Big Bang model are therefore mainly aimed at describing different phases of the carcinogenic process, with epiTOC providing an approximate measure of cell division numbers per stem cell before the first cancer clone emerges and with the Big Bang model providing a description of subsequent cancer evolution. Both models together are highly consistent with an overarching “phase transition” model of oncogenesis, in which epigenetic clonal mosaicism is maximal before cancer emerges [61].

## Conclusions

The epigenetic mitotic clock-like signature presented here exhibits a consistent universal pattern of acceleration in cancer, in precancerous epithelial lesions, and in normal epithelial cells exposed to a major carcinogen. We propose that DNAm-based models such as epiTOC may constitute informative biomarkers of cancer risk if evaluated in the cell of origin or in a relevant surrogate tissue.

## Methods

### Construction of an epigenetic mitotic clock (epiTOC): a mathematical model

In order to provide the rationale for the procedure of constructing epiTOC, as described in the next subsection, we here first present the salient features of the underlying mathematical model. We assume that cancer risk of a tissue type *t* in an individual *s*, which we denote as *CR(s*,*t)*, is a monotonically increasing function *f* of the total number of stem cell divisions (per stem cell) incurred in the tissue, i.e., we assume that:

$$ CR\;\left(s,t\right)=f\left[ TNSC\left(s,t\right)\right] $$

where *TNSC* is the total number of stem cell divisions, which will depend on tissue type *t* and individual *s*. We assume further that *TNSC* can be approximated as:

1 $$ TNSC\left(s,t\right)=A(s)\left[IR(t) + E(s)ER(t)\right]=A(s)IR(t) + A(s)E(s)ER(t) $$

where *A(s)* denotes the chronological age of the individual *s*, *IR(t)* denotes the intrinsic rate of stem cell divisions per stem cell in tissue type *t*, *E(s)* is a complex non-linear (generally unknown) positively valued function representing the exposure of individual *s* to a cancer risk factor, and where *ER(t)* denotes an extrinsic rate of stem cell division associated with exposure to the cancer risk factor and which we assume may depend on tissue type *t.* Note that we assume that the intrinsic rate *IR* only depends on tissue type and that it is, therefore, independent of chronological age and individual *s*, so we are ignoring genetic factors which may influence the rate of stem cell division.

Motivated by previous work [4], we further assume that specific CpG sites in the genome acquire stochastic DNAm errors during cell replication and that the cumulative number of DNAm errors is a linear function of the total number of stem cell divisions per stem cell. We denote the cumulative amount of DNAm errors as “*pcgtAge*” in anticipation of how the corresponding CpGs will be identified (see the “Construction of an epigenetic mitotic-like clock: CpG selection” section below). Hence, we can also write a linear model of the form:

2 $$ pcgtAge\left(s,t\right) = \alpha (t) + \xi (t)\  TNSC\left(s,t\right) + \varepsilon $$

However, we can’t train a DNAm-based model with *TNSC*, since the latter depends on the exposures which are generally unknown. Instead, we could link Eqs. 1 and 2 above to train a DNAm-based model. Since *IR* is dependent on the tissue type, and since *E(s)* and *ER(t)* are generally unknown quantities, it is best to focus on one tissue type only and to consider a healthy population of individuals. This then allows us to assume that *E(s) ≈ 0* and that *IR(t) = constant*, so, to a first approximation:

$$ \begin{array}{c} pcgtAge\left(s,t\right) = \alpha (t) + \xi (t)\ A(s)IR(t) + \varepsilon \\ {} = \alpha (t) + \gamma (t)\ A(s) + \varepsilon \end{array} $$

where we have absorbed the term *IR* into a new slope coefficient. Thus, to identify CpGs whose DNAm levels correlate with *TNSC*, it is justified to correlate DNAm to chronological age, as long as we use one tissue type and focus on healthy individuals. However, since many biological processes may be associated with distinct age-associated DNAm changes, it is necessary to make a selection of CpGs which are more likely to capture the DNAm aberrations caused by cell division.

### Construction of an epigenetic mitotic-like clock: CpG selection

Motivated by the above mathematical model, we posited that we could identify relevant CpGs as follows: (i) identify CpG sites undergoing age-associated DNAm changes in a large DNAm dataset of healthy individuals, encompassing one tissue type only and correcting for potential changes in cell type composition; (ii) identify a subset of these that map to PCGT promoters, i.e., marked by the PRC2 complex, and which are constitutively unmethylated in a ground state of age zero (e.g., fetal tissue).

To justify (i), we reasoned that using multiple tissues, which would naturally differ in their mitotic tick rates, would only hamper or confound derivation of a mitotic clock (see above mathematical model). Correction for underlying changes in cell type composition is, however, an important potential confounder if we are to use only one tissue type. For these reasons, we used the dataset of 656 whole blood samples from Hannum et al. [28], representing one of the largest cohorts of healthy individuals which have been profiled with Illumina 450 k DNAm beadarrays, and a tissue type (blood) for which accurate correction for changes in blood cell type composition is possible [62]. We justify (ii) on grounds that a recent study has shown that DNAm changes occurring during hematopoietic ontogeny involve preferentially DNAm increases at PCGT promoters, i.e., sites marked by the PRC2 complex [26]. Thus, we reasoned that focusing on a subset of such promoter CpGs which are also constitutively unmethylated in a large set of fetal tissues [63] would provide us with the right markers to measure the rate of cell division.

In detail, using Hannum’s whole blood samples, we ran linear regressions of chronological age versus DNA methylation beta profiles adjusted for plate, sex, and estimates of blood cell subtypes. Estimates of blood cell subtypes were obtained using quadratic programming [64] and a novel blood cell subtype DNAm reference matrix (Additional file 1) constructed by integrating the Illumina 450 k DNAm data from Reinius et al. [65] with blood cell subtype-specific DNase hypersensitive site data from the NIH Epigenomics Roadmap (Teschendorff A et al: A comparison of reference-based algorithms for correcting cell-type heterogeneity in Epigenome-Wide Association Studies, submitted). Age-associated CpGs were selected at a false discovery rate threshold of <0.05. Subsequently, these age-CpGs were filtered for those mapping unambiguously to within 200 bp of a transcription start site (TSS200 probes). We note that this restriction to TSS200 probes was done in order to minimize differences in the ground state (i.e., at age zero) methylation levels between probes, which facilitates the later construction of the *pcgtAge* score as an average of the probes. With this restriction to TSS200 probes as well as the inherent restricted coverage of the 450 k beadarrays, we nevertheless still covered 72 % of all PCGT promoters as defined in Lee et al. [66]. The age-associated TSS200 CpGs were then divided into age-hypermethylated CpGs and age-hypomethylated ones. Age-hypermethylated CpGs were filtered further, selecting only those with absent or low (beta <0.2) methylation across 52 fetal tissue samples encompassing 11 tissue types (cord blood (GSE72867), stomach, heart, tongue, kidney, liver, brain, thymus, spleen, lung, adrenal gland [63]). These unmethylated promoter CpGs were divided further into those marked by PRC2 in human embryonic stem cells (hESCs) and those that are not, according to the annotation provided [66]. This resulted in an age-hypermethylated set of 385 CpGs, which we denote “*pcgtAge*”. In the case of the age-hypomethylated promoter CpGs, we selected those with a methylation level of at least 0.3 across all 52 fetal tissue samples in order to guarantee that the observed hypomethylation at these sites is genuine and of potential biological significance. This resulted in a second set of 656 CpGs, denoted “hypoAge”.

Age-correlative DNAm deviation scores, *pcgtAge* and *hypoAge*, were then calculated as the average DNAm over the respective CpG sites. Mathematically, *pcgtAge* for sample *s* in tissue type *t*, is calculated as:

$$ pcgtAge\left(s,t\right)=\frac{1}{n}{\displaystyle {\sum}_{c=1}^n{\beta}_{cst}} $$

By construction, this score should correlate with chronological age, and we can estimate parameters *α’* and *γ’* from fitting a linear model:

$$ pcgtAge\left(s,t\right)\kern0.5em =\kern0.5em \alpha (t)\kern0.5em +\kern0.5em \gamma (t)\kern0.5em A(s)+\kern0.5em \varepsilon ' $$

From this, we can now obtain an estimate of *TNSC(s*,*t)* for a sample of the same tissue type *t* but which is not healthy, e.g., one exposed to a cancer risk factor:

3 $$ TNSC\left(s,t\right)=\left[ pcgtAge\left(s,t\right)\kern0.5em \hbox{--} \kern0.5em \alpha '(t)\right]IR(t)/\kern0.5em \gamma '(t) $$

since estimates for *IR(t)* are available from the literature [2]. Thus, for the same tissue type *t* used in the training (i.e., blood) and from which the estimates *α’* and *γ’* were obtained, we can derive estimates of *TNSC* but not so for a different tissue type (e.g., lung or breast). However, the second term in Eq. 3 above is independent of the sample *s*; hence, one can write for the cancer risk:

$$ CR\left(s,x\right)=f\left[ TNSC\left(s,x\right)\right]=f\left[ pcgtAge\left(s,x\right)IR(t)/\ \gamma '(t)\kern0.5em \hbox{--} \kern0.5em  term(t)\right] $$

and so, if *pcgtAge(s*_*1*_,*x) > pcgtAge(s*_*2*_,*x)*, then the cancer risk (*CR*) of sample *s*_*1*_ is also greater than that of *s*_*2*_: *CR(s*_*1*_,*x) > CR(s*_*2*,_*x)*. Hence, a higher *pcgtAge* score should be indicative of a higher mitotic tick rate and indicate a higher cancer risk, which we can formally test if data for pre-cancerous lesions are available. Similarly, a lower *hypoAge* score would correspond to a higher cancer risk.

From Hannum et al. [28] whole blood data set, we estimated for the *pcgtAge* model that *α*’ = 0.052 and *γ’* = 0.000345. From the 81 normal breast tissue samples from TCGA, we estimated *α*’ = 0.053 and *γ’* = 0.000165, and from the combined 73 normal lung samples from the TCGA we estimated *α*’ = 0.021 and *γ’* = 0.000588.

We note that even in the absence of an *IR(t)* estimate one can still estimate the relative *TNSC* numbers of two samples *s*_*1*_ and *s*_*2*_ of the same tissue type since the ratio:

$$ TNSC\left({s}_1,\ t\right)\kern0.5em /\kern0.5em  TNSC\left({s}_{2, }t\right)\kern0.5em =\kern0.5em \left[ pcgtAge\left({s}_1,t\right)\ \hbox{--}\ \alpha '(t)\right]\kern0.5em /\kern0.5em \left[ pcgtAge\left({s}_2,t\right)\kern0.5em \hbox{--} \kern0.5em \alpha '(t)\right] $$

does not depend on *IR(t)*.

### Validation of the age-correlative models in blood and other normal tissue types

EpiTOC and the analogous model based on age-hypomethylated sites were tested in the large independent whole blood Illumina 450 k dataset of Liu et al. [31] using only healthy controls (over 300 samples). We also tested these two models in other normal tissue types. For this purpose, we used normal-adjacent tissue from TCGA, focusing on tissues for which there were enough normal samples and for which there had been corresponding fetal tissue used in the derivation and selection of the CpGs making up these models. This included 38 normal colon (normal-adjacent tissue to colon adenocarcinoma (COAD)), 160 normal kidney (adjacent to kidney renal cell carcinoma (KIRC)), 47 normal liver (adjacent to liver hepatocellular carcinoma (LIHC)), and 73 normal lung samples (adjacent to lung squamous cell carcinoma (LSCC)/ lung adenocarcinoma (LUAD)).

### Validation of the epigenetic mitotic clock in normal tissue

In order to demonstrate that our age-correlative models define approximate mitotic clocks, i.e., that the relation *TNSC(s*,*t) ~ pcgtAge(s*,*t)* holds, we estimated the age-correlative scores in the normal tissue samples of TCGA [37], for which estimates of the intrinsic stem cell division rates (*IR*) per stem cell and per year are available from [2]. Specifically, this included colon (*IR* = 73 divisions per stem cell per year), rectum (*IR* = 73), esophagus (*IR* = 17.4), head and neck (*IR* = 21.5), liver (*IR* = 0.9125), lung (*IR* = 0.07), pancreas (*IR* = 1) and thyroid (*IR* = 0.087), encompassing a total of 288 normal tissue samples. Cumulative total number of cell divisions per stem cell (i.e., *TNSC)* was estimated for each of these 288 normal “healthy” samples as the product of chronological age (tissue-independent) and the corresponding rate *IR* (tissue-dependent), i.e., as *TNSC(s*,*t) = A(s)IR(t)*. The sample-specific scores *pcgtAge*, *nonpcgtAge*, and *hypoAge* were then correlated to these sample-specific cumulative cellular turnover rates using a linear regression framework adjusted for chronological age (in order to avoid the expected trivial correlation by age).

### Validation of the epigenetic mitotic clock in cancer tissue: construction of an mRNA expression based-mitotic index

Because cell division rates are altered in cancer, we validated the mitotic nature of the age-correlative scores *pcgtAge* and *hypoAge* in cancer samples by comparison of these scores to an mRNA expression-based mitotic index. This mitotic index was constructed by first taking the interConstruction of the epiTOC model of genes in the cell proliferation cluster of Ben-Porath et al. [67] and those of the proliferation signature of Rhodes et al. [68]. This resulted in nine genes (*CDKN3*, *ILF2*, *KDELR2*, *RFC4*, *TOP2A*, *MCM3*, *KPNA2*, *CKS2*, and *CDC2*). The mitotic index was then defined as the average mRNA expression over these nine genes. This mRNA expression-based mitotic index was validated in 15 cancer types of TCGA by demonstrating that it is significantly increased in each cancer type compared to its corresponding normal tissue type. We verified that it was a more reliable mitotic index than *PCNA* expression (not shown).

### Cancer and pre-cancerous Illumina 450 k datasets

We downloaded and processed level 3 Illumina 450 k and RNA-SeqV2 data from TCGA [37], as described by us previously [69]. In total, we considered 15 cancer types: BLCA (bladder adenocarcinoma, nN = 19, nC = 204), BRCA (breast adenocarcinoma, nN = 81, nC = 652), COAD (colon adenocarcinoma, nN = 38, nC = 272), ESCA (esophageal carcinoma, nN = 15, nC = 126), HNSC (head and neck squamous cell carcinoma, nN = 45, nC = 402), KIRC (kidney renal cell carcinoma, nN = 160, nC = 299), KIRP (kidney renal papillary carcinoma, nN = 45, nC = 196), LIHC (liver hepatocellular carcinoma, nN = 47, nC = 176), LSCC (lung squamous cell carcinoma, nN = 41, nC = 275), LUAD (lung adenomacarcinoma, nN = 32, nC = 399), PAAD (pancreatic adenoma carcinoma, nN = 10, nC = 146), PRAD (prostate ademoma carcinoma, nN = 48, nC = 278), READ (rectal adenoma carcinoma, nN = 7, nC = 95), THCA (thyroid carcinoma, nN = 53, nC = 489), and UCEC (uterine cervix endometrial carcinoma, nN = 34, nC = 374).

We used Illumina 450 k DNAm data from the three previous publications and which had profiled precursor cancer lesions or normal-adjacent tissue in addition to normal samples. Briefly, these datasets were: (i) a dataset of normal lung and lung carcinoma in situ (LCIS) samples, with a subset of these progressing to an invasive lung cancer, previously described in [20]; (ii) a dataset of normal breast tissue and ductal carcinoma in situ (DCIS) samples, with a subset of these progressing to an invasive breast cancer, previously described in [40]; (iii) a dataset of 50 normal breast tissue samples, 42 matched normal-adjacent breast tumor pairs, and an additional 263 breast cancers, previously described in [41].

Age-correlative scores *pcgtAge* and *hypoAge* were estimated as average DNAm levels over the corresponding CpG sites in all of these samples.

### Buccal tissue Illumina 450 k set

Illumina 450 k DNAm profiles were generated for buccal samples from 790 women, all aged 53 at the time of sampling, as described by us previously [20]. For a subset of 152 women, there were matched buccal–blood samples. We used the normalized data as used in our previous publication.

### Non-TCGA cancer tissue and ENCODE cell line DNAm datasets

Illumina 450 k DNAm data for 32 glioblastoma multiformes (GBM) were downloaded from the Gene Expression Omnibus (GEO; accession number GSE30338) [70] and normalized with BMIQ. Illumina 450 k data for 215 ovarian cancers was processed and normalized with BMIQ as described by us previously (GEO: GSE74845) [71]. Illumina 27 k DNAm data for a total of 49 cervical cancer epithelial samples were processed and normalized as described by us previously (GEO: GSE30759) [15]. Cell line Illumina 450 k DNAm data for 62 cell lines was obtained from ENCODE via GEO (GSE40699). These data were subsequently normalized with BMIQ.

### Purified T-cell, B-cell and monocyte Illumina 450 k sets

Illumina 450 k profiling was performed on 100 purified CD19+ B-cell samples, 98 CD4+ T-cell samples, and 104 CD14+/CD16− monocytes from a total of 52 monozygotic twins discordant for type 1 diabetes, as described by us previously (Paul D et al: Increased DNA methylation variability in type 1 diabetes across three immune effector cell types, submitted) Here we only used the samples from the healthy controls, amounting to 50 B-cell, 49 T-cell, and 52 monocyte samples. Across all cell types, the mean cell purity was 90 %. The Illumina 450 k data were processed as described (Paul D et al: Increased DNA methylation variability in type 1 diabetes across three immune effector cell types, submitted) and are available from the European Genome-phenome Archive (EGA; https://www.ebi.ac.uk/ega/) under accession number EGAS00001001598.

In addition, we used Illumina 450 k data of an independent set of purified CD4+ T-cell (n = 214) and monocyte (n = 1202) samples, as generated by the MESA study [33]. These data were downloaded from the GEO (GSE56046 and GSE56581). Intra-array normalization was performed with BMIQ.

### Stem cell Illumina DNAm sets

We downloaded normalized Illumina 27 k data for two sets of stem cell-like cell populations. One set had profiled eight mesenchymal stem cell (MSC) samples (all of the same low passage number of 2) collected from the bone marrow of eight donors of widely different ages [35]. Data were obtained from the GEO (GSE17448). Another set consisted of 12 CD34+ hematopoetic progenitor cell (HPC) samples collected from either cord blood (n = 7) or mobilized peripheral blood from adults (n = 5, age range 28 to 41 years) [36]. Data were obtained from EBI’s ArrayExpress repository (E-MTAB-487).

### Software availability

An R script implementing epiTOC and the associated probe IDs of the CpGs making up epiTOC is available as Additional files 5 and 6.

## Additional files

Additional file 1: Blood reference DNA methylation data matrix in Excel format. Detailed legend in file. (XLS 85 kb) Additional file 2: The 385 PCGT CpGs that make up epiTOC. Detailed legend in file. (XLS 205 kb) Additional file 3: Supplementary information document with all supplementary figures and their legends. (PDF 1902 kb) Additional file 4: Gene set enrichment analysis results (in Excel table format) of the 385 PCGT CpGs that make up epiTOC. Columns label the biological term, number of genes present in the term, number and corresponding fraction of genes present on the bead array, the number in the overlap between term and the genes mapped by the 385 PCGT CpGs, the odds ratio (OR), the Fisher test one-tailed *P* value of enrichment, the adjusted *P* value (Benjamini–Hochberg adjusted) and the overlapping gene symbols. (XLS 1403 kb) Additional file 5: epiTOCcpgs.RData. An R object data file containing the 450 k probe IDs of the 385 PCGT CpGs that make up epiTOC. (RDATA 1 kb) Additional file 6: EstEpiTOC.R. An executable R script function to allow estimation of the *pcgtAge* score in independent samples. (R 1 kb)

## Abbreviations

AUC: area under the curve
CI: confidence interval
DCIS: ductal carcinoma in situ
DNAm: DNA methylation
EGA: European Genome-phenome Archive
GEO: Gene Expression Omnibus
hESC: human embryonic stem cell
HPC: hematopoietic progenitor cell
IBC: invasive breast cancer
ILC: invasive lung cancer
LCIS: lung carcinoma in situ
MSC: mesenchymal stem cell
PCGT: Polycomb group target
SPY: smoking pack-year
TCGA: The Cancer Genome Atlas
TNSC: total number of stem cell divisions per stem cell.
