# Supplementary material for: Correlation of an epigenetic mitotic clock with cancer risk
Source: Genome Biol. 2016 Oct 3;17:205. doi: 10.1186/s13059-016-1064-3 (PMC5046977; doi:10.1186/s13059-016-1064-3)
Supplement: Additional file 3: — Supplementary information document with all supplementary figures and their legends. (PDF 1902 kb) [file 13059_2016_1064_MOESM3_ESM.pdf]

Additional File 3-Supplementary Information

Correlation of an epigenetic mitotic clock with cancer risk

Zhen Yang [1,7], Andrew Wong [2] , Diana Kuh [2], Dirk S. Paul [3], Vardhman K. Rakyan [4], R David Leslie [4], Shijie C Zheng [1], Martin Widschwendter [5], Stephan Beck [3] and Andrew E. Teschendorff [1,5,6,7,*]

1. CAS Key Laboratory of Computational Biology, CAS-MPG Partner Institute for Computational Biology, 320 Yue Yang Road, Shanghai 200031, China.
2. MRC Unit for Lifelong Health and Ageing at University College London, London, United Kingdom.
3. Medical Genomics, UCL Cancer Institute, University College London, 72 Huntley Street, London WC1E 6BT, United Kingdom.
4. The Blizard Institute, Barts and The London School of Medicine and Dentistry, Queen Mary University of London, London E1 2AT, UK.
5. Department of Women's Cancer, University College London, 74 Huntley Street, London WC1E 6AU, United Kingdom.
6. Statistical Cancer Genomics, Paul O'Gorman Building, UCL Cancer Institute, University College London, 72 Huntley Street, London WC1E 6BT, United Kingdom.
7. Equal Contribution.

*Corresponding author: Andrew E. Teschendorff- a.teschendorff@ucl.ac.uk , andrew@picb.ac.cn

DESCRIPTION

This Supplementary Information document contains all Supplementary Figures and their legends.

SUPPLEMENTARY FIGURES

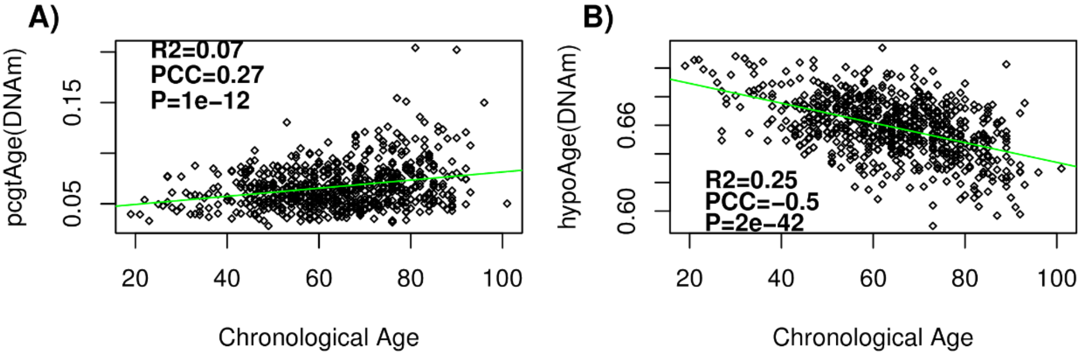

fig.S1. Age-correlative models in the Hannum et al discovery set. A) The pcgtAge score (y-axis) against chronological age (x-axis) of the whole blood sample. The pcgtAge score is the average DNAm over 385 promoter PCGT CpGs, all unmethylated across 11 fetal tissue types and exhibiting a significant (FDR<0.05) age-associated hypermethylation after adjustment for changes in blood-cell type composition**.** R^2, Pearson Correlation Coefficient (PCC) and linear regression P-value is given. **B)** As A), but now for a model based on 656 promoter CpGs, all exhibiting partial methylation (beta>0.3) across all fetal tissue types, and exhibiting significant (FDR<0.05 after adjustment for blood cell type composition) age-associated hypomethylation. Illumina 450k data used is from Hannum et al (Hannum et al., 2013).

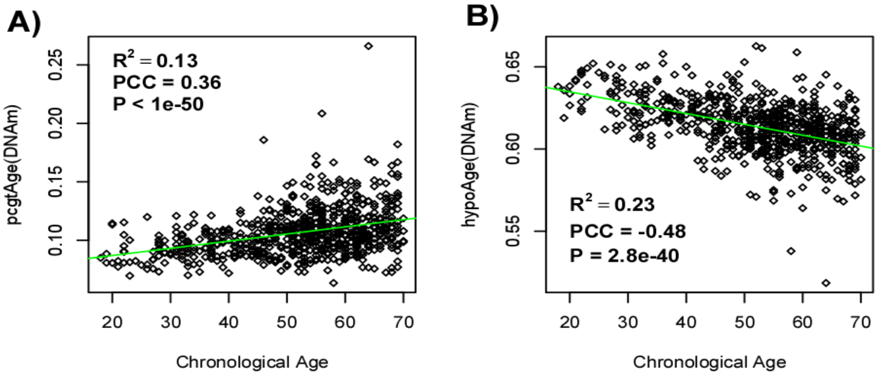

fig.S2. Validation of age-correlative models in the Liu et al set. A) Model based on the age-hypermethylated PCGT CpGs, **B)** Model based on the age hypomethylated CpGs. Illumina 450k data used are the control samples from Liu et al (Liu et al., 2013).

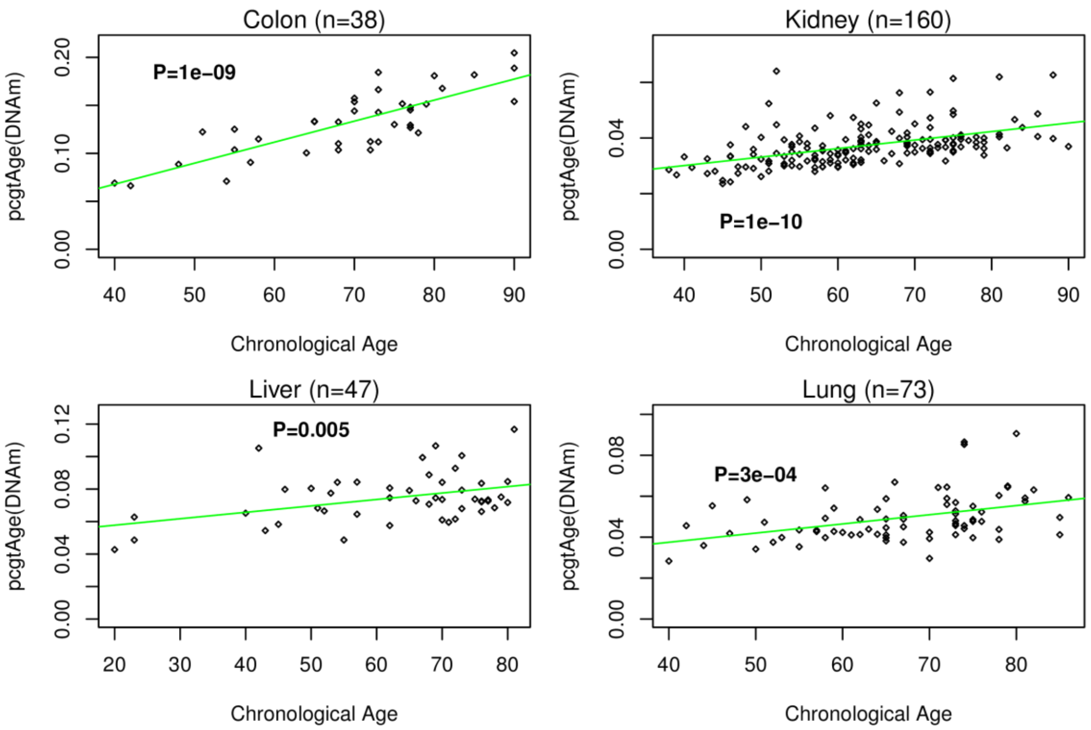

fig.S3. Validation of epiTOC (pcgtAge) age-correlative model in normal tissue sets. Scatterplots of pcgtAge (y-axis) vs chronological age for 4 normal tissue types collected from the TCGA. P-value is from a linear regression. We show this for tissues for which there were enough normal samples, and for which there was corresponding fetal tissue when deriving the age-hypermethylated PCGT CpGs.

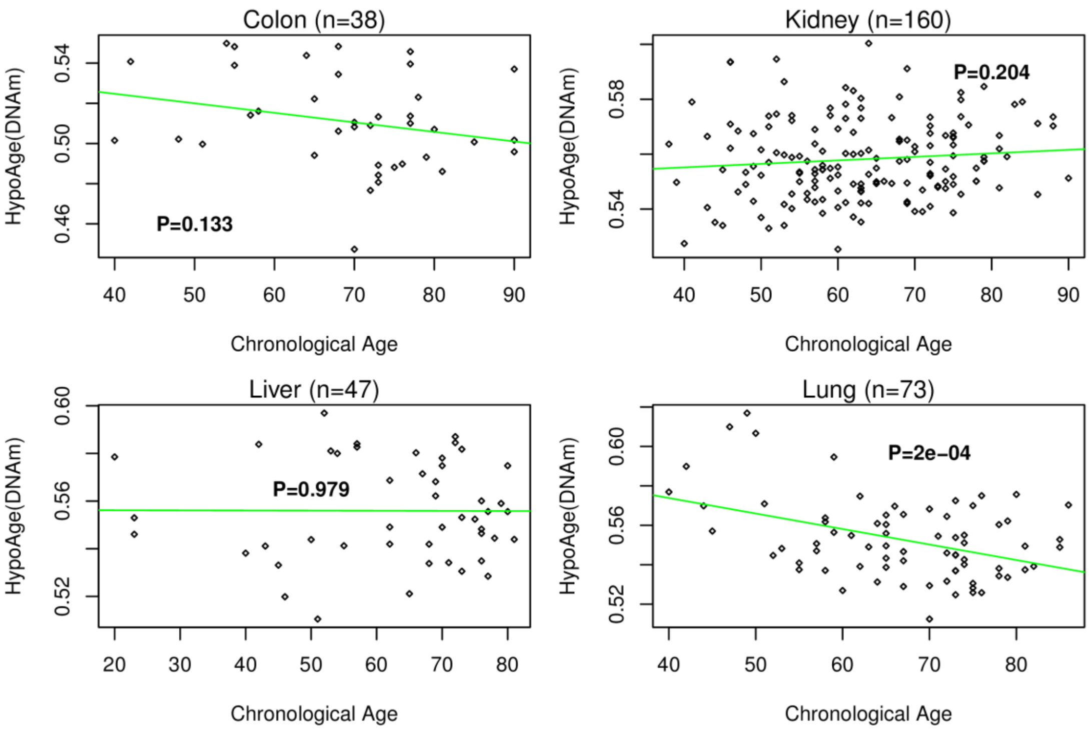

fig.S4. Age-hypomethylated model does not validate in all normal tissue sets. Scatterplots of HypoAge (y-axis) vs chronological age for 4 normal tissue types from the TCGA. P-value is from a linear regression. We show this for tissues for which there were enough normal samples, and for which there was corresponding fetal tissue when deriving the age-hypomethylated promoter CpGs.

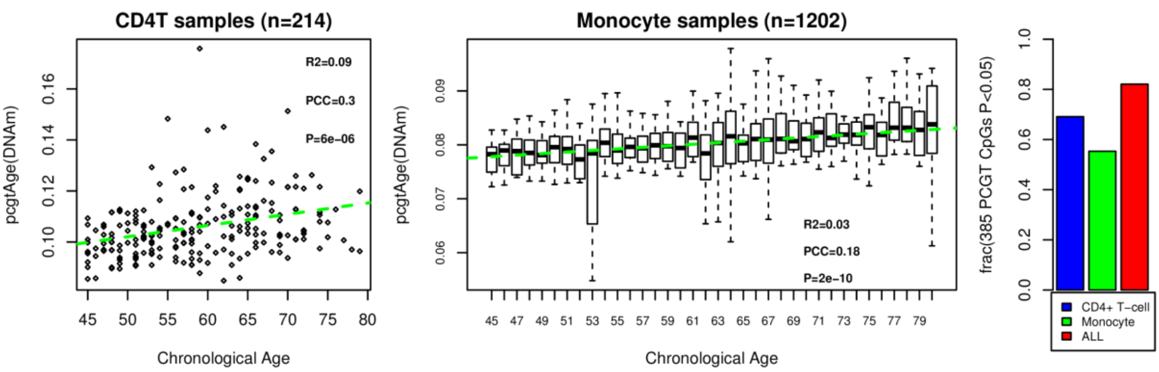

fig.S5. Correlation of pcgtAge score with chronological age in two large purified cell sets. Scatterplots of pcgtAge vs chronological age for two purified cell Illumina 450k sets (CD4+ T-cells and Monocytes) from Reynolds et al (Reynolds et al., 2014). Number of samples is given above plots. Green dashed line represents the linear least squares fit. R^2 values, Pearson Correlation Coefficients (PCC) and linear regression P-values are given. Note that although large, these sample sets are from older cohorts. Barplot comparing the fraction of the 385 PCGT CpGs of epiTOC which are significantly hypermethylated with age in each of these two purified sample sets, as well as the fraction of epiTOC CpGs which are significantly hypermethylated with age in at least one of these studies (“ALL”-red).

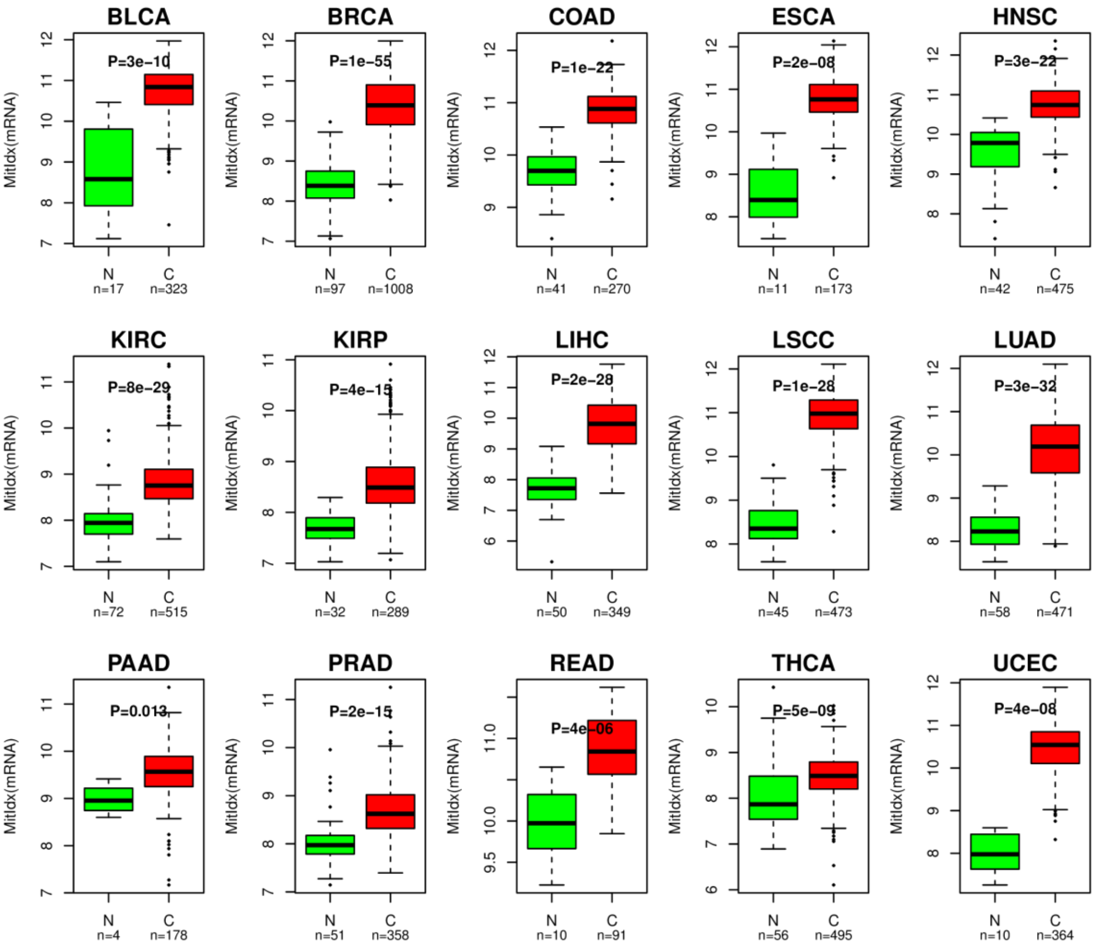

fig.S6. Validation of mRNA expression based mitotic index in cancer (all samples). Boxplots compare the mRNA expression-based mitotic index (y-axis) between each normal and cancer group for 15 TCGA cancer types, as indicated. P-value is from a one-tailed Wilcoxon rank sum test to confirm that the index is higher in cancer. The mitotic index was constructed as the average mRNA expression over 9 genes (*CDKN3, ILF2, KDELR2, RFC4, TOP2A, MCM3, KPNA2, CKS2 and CDC2*), which belong to both the cell proliferation cluster of (Ben-Porath et al., 2008) and to the proliferation signature of (Rhodes et al., 2004). This combined index performed better than *PCNA* expression (not shown).

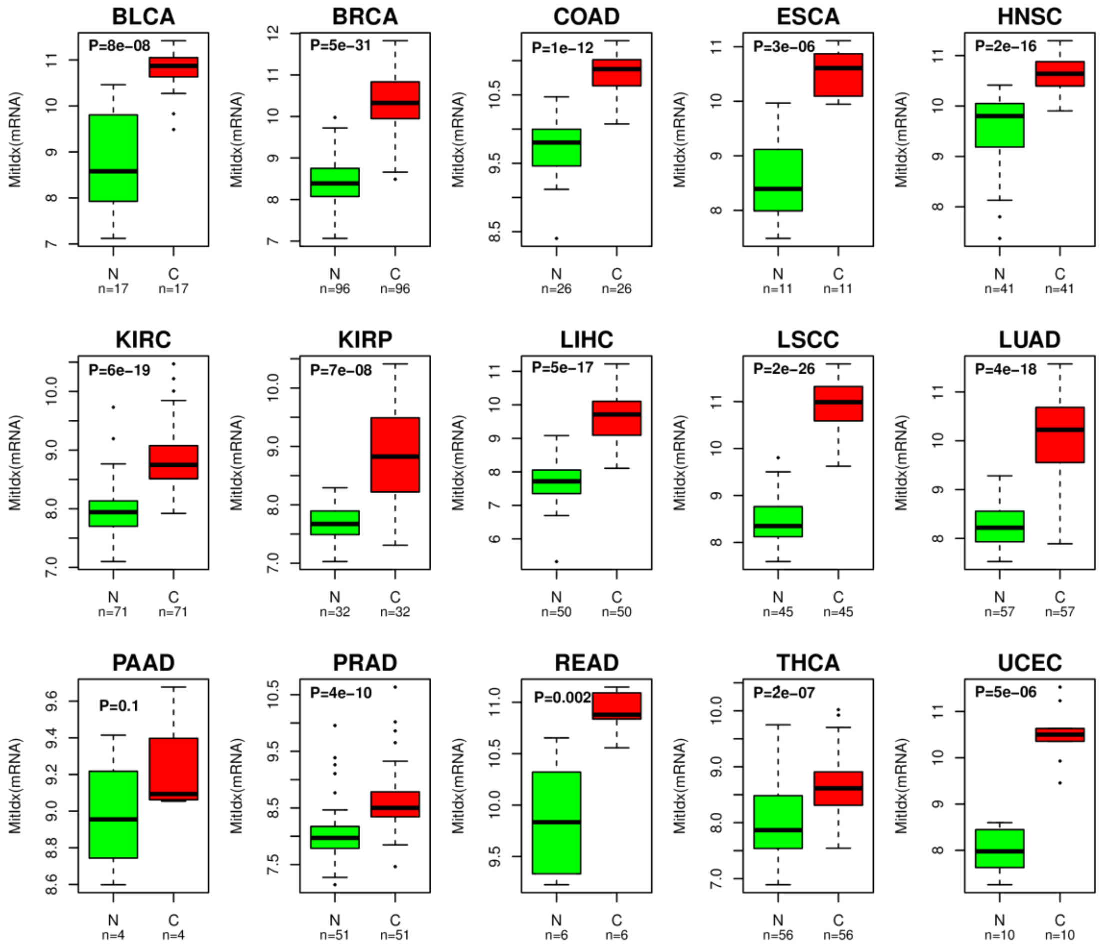

fig.S7. Validation of mRNA expression based mitotic index in cancer (using only matched normal-tumor pairs, hence age-matched). Boxplots compare the mRNA expression-based mitotic index (y-axis) between each normal and cancer group for 15 TCGA cancer types, as indicated, and using only age-matched normal-tumor pairs. P-value is from a one-tailed Wilcoxon rank sum test to confirm that the index is higher in cancer. The mitotic index was constructed in fig.S6.

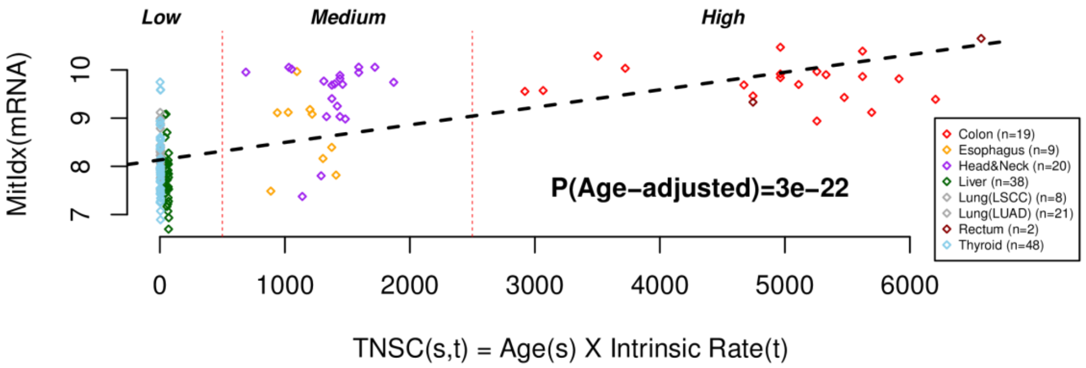

fig.S8. Correlation of the mRNA-expression based mitotic index with TNSC across normal tissues of the TCGA. The x-axis labels the cumulative total number of stem-cell divisions (TNSC) incurred per stem-cell in the given normal tissue This number is the product of the intrinsic rate of cell-divisions per year per stem-cell (tissue-dependent) with the chronological age of the sample (tissue-independent). The y-axis labels the expression-based mitotic index of the sample. Samples have been colored according to the normal tissue of origin (normal samples adjacent to TCGA cancer types, as indicated). Number of normal samples for each tissue is given. P-value from a linear regression adjusted for chronological age is given.

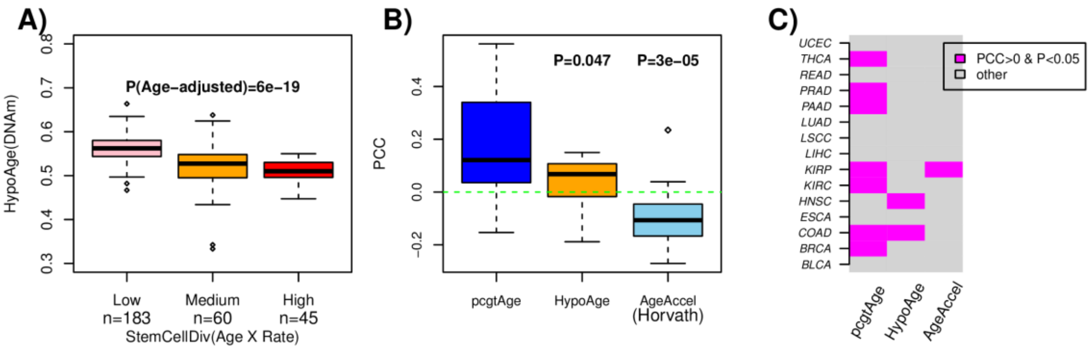

fig.S9. A) The estimated HypoAge-score against cellular turnover group. Number of samples in each group is given. P-value is from a linear regression adjusted for chronological age of samples. **B)** Boxplots of the Pearson Correlation Coefficient (PCC) of pcgtAge, HypoAge and Horvath's age-acceleration measure, with an mRNA expression based mitotic index, as estimated over all cancer samples of a given cancer type for a total of 15 different TCGA cancer types. Thus, each boxplot has 15 data points. P-values are from a one-tailed paired Wilcoxon rank sum test comparing pcgtAge to each of the other categories. Note that in the case of HypoAge, we used the negative of the PCC since the expected correlation with the mitotic index is negative given that it measures hypomethylation. **C)** Corresponding heatmap indicating for which cancer-types there was a significant positive PCC between pcgtAge and

Horvath's AgeAccel with the mRNA expression based mitotic index. Note that in the case of HypoAge, we consider significant negative PCC values.

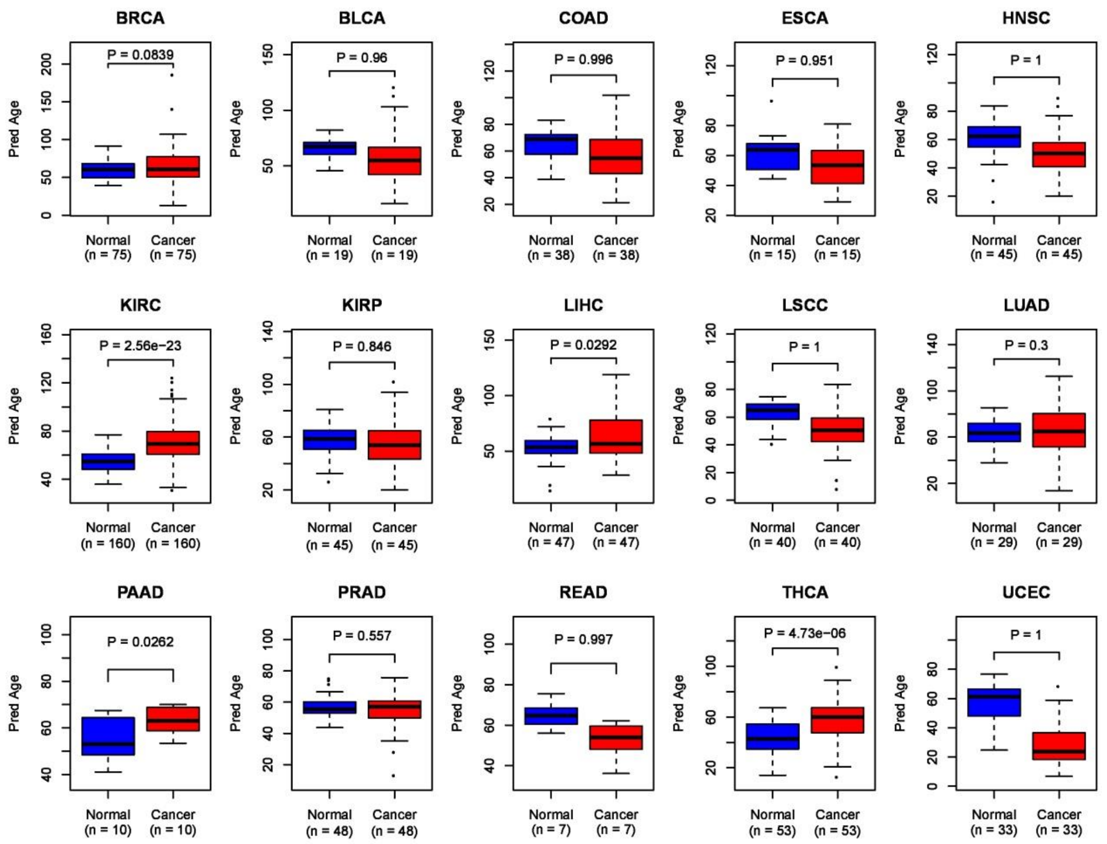

fig.S10. DNA methylation age as predicted using Horvath's epigenetic clock does not present a consistent age-acceleration pattern across cancer types. Only age-matched normal-tumor pairs were considered for each of 15 different TCGA cancer types as indicated. P-values are from one tailed Wilcoxon rank sum test.

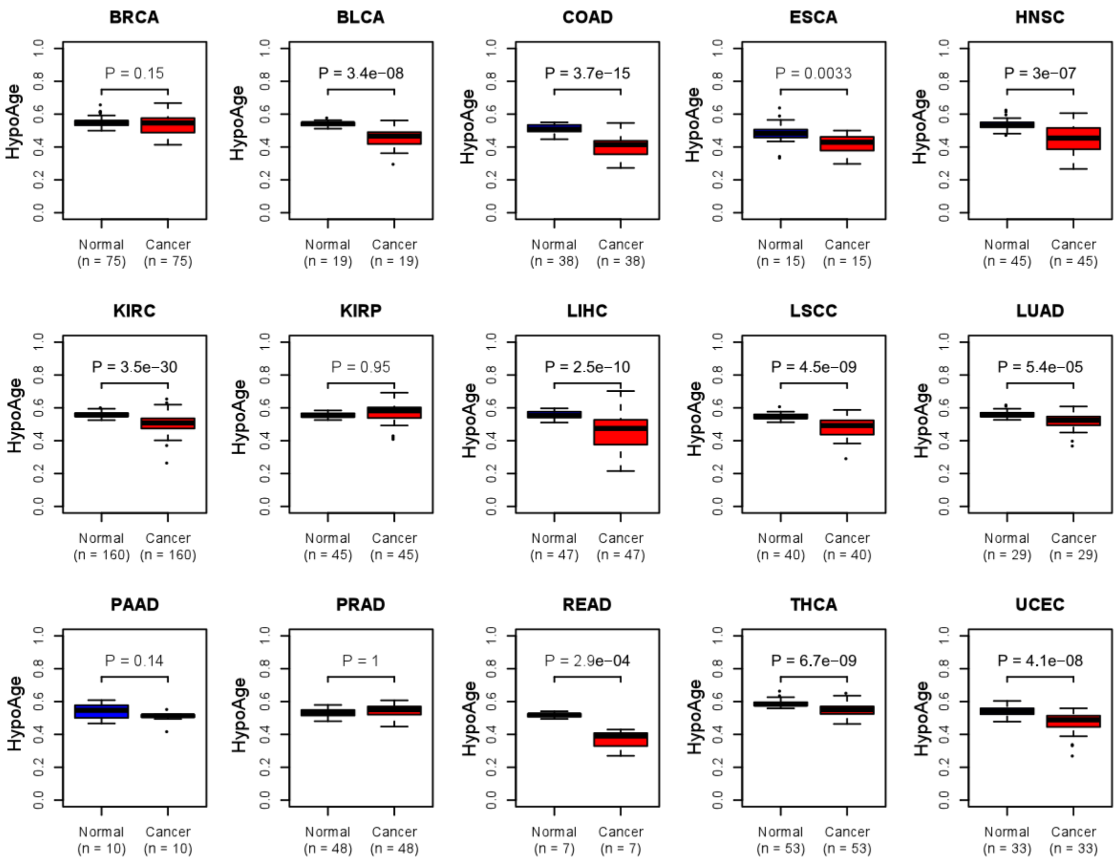

fig.S11. The age-hypomethylated based age-correlative model does not predict age-acceleration in all cancer types. Only age-matched normal-tumor pairs were considered for each of 15 different TCGA cancer types as indicated. P-values are from one tailed Wilcoxon rank sum test. Note that here, because we are considering hypomethylation, “acceleration” means a lower HypoAge score in cancer compared to normal. So, for 4 cancer types (BRCA, KIRP, PAAD, PRAD) one does not observe acceleration in cancer.

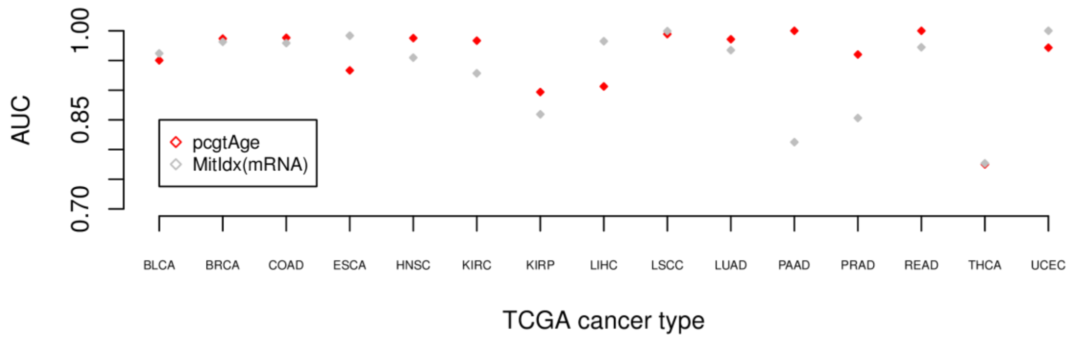

fig.S12. Comparison of discrimination accuracy (AUC) of normal and cancer tissue between pcgtAge and the mRNA-based mitotic index. Only age-matched normal-tumor pairs were considered for each of 15 different TCGA cancer types to estimate the Area under the curve (AUC, y-axis).

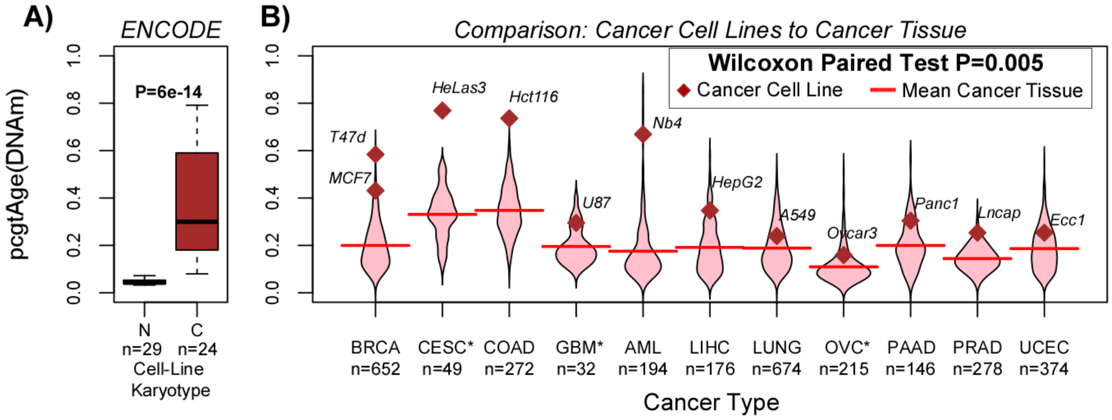

fig.S13. Increased *pcgtAge* score in cancer cell lines. A) Comparison of *pcgtAge*-score of ENCODE cell-lines of both normal (N) and cancer (C) karyotypes. P-value is from a one-tailed Wilcoxon rank sum test. **B)** Comparison of the *pcgtAge*-score for 11 cancer-cell lines (from ENCODE) to their respective cancer tissue. *indicates cancer-types for which the Illumina 450k data was not derived from the TCGA. BRCA=breast cancer, CESC=cervical squamous carcinoma, COAD=colon adenoma carcinoma, GBM= glioblastoma multiforme, AML=acute myeloid leukemia, LIHC=liver hepatocellular carcinoma, LUNG=lung squamous and lung adenoma carcinoma, OVC=ovarian cancer, PAAD= pancreatic adenoma carcinoma, PRAD=prostate adenoma carcinoma, UCEC= uterine cervix endometrial carcinoma. P-value shown is from a paired Wilcoxon rank sum test comparing the cancer cell-lines to the average value for each cancer tissue.

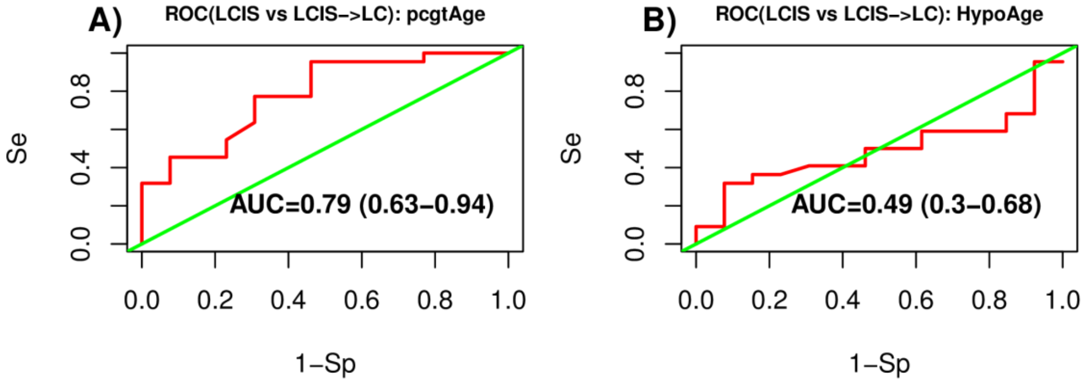

fig.S14. pcgtAge correlates with prospective lung cancer risk whereas the age-hypomethylated based model does not. A) ROC-analysis testing whether pcgtAge discriminates LCIS that progress to invasive lung cancer from those which do not. AUC and 95%CI are given. **B)** As A), but now for the HypoAge score derived from the age-hypomethylated based model.

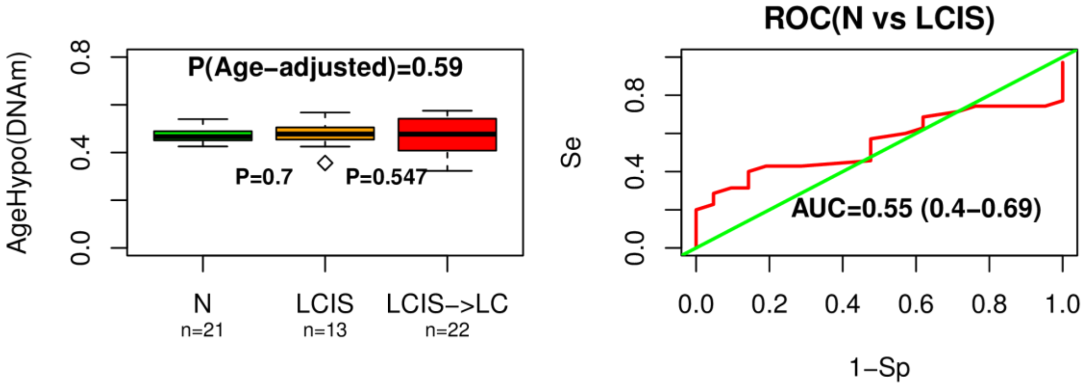

fig.S15. Age-hypomethylated based model does not predict age-acceleration in LCIS. Boxplot comparing HypoAge-score for normal lung tissue (N), lung carcinoma in situ (LCIS) and LCIS samples that progressed to an invasive lung cancer (LC). Number of samples in each group given below boxplot. One-tailed Wilcoxon rank sum test between the neighboring groups are given, as well as the P-value from a linear regression of HypoAge vs. group, adjusted for age. Right panel is the ROC for discriminating normal from all LCIS samples. AUC and 95%CI are given.

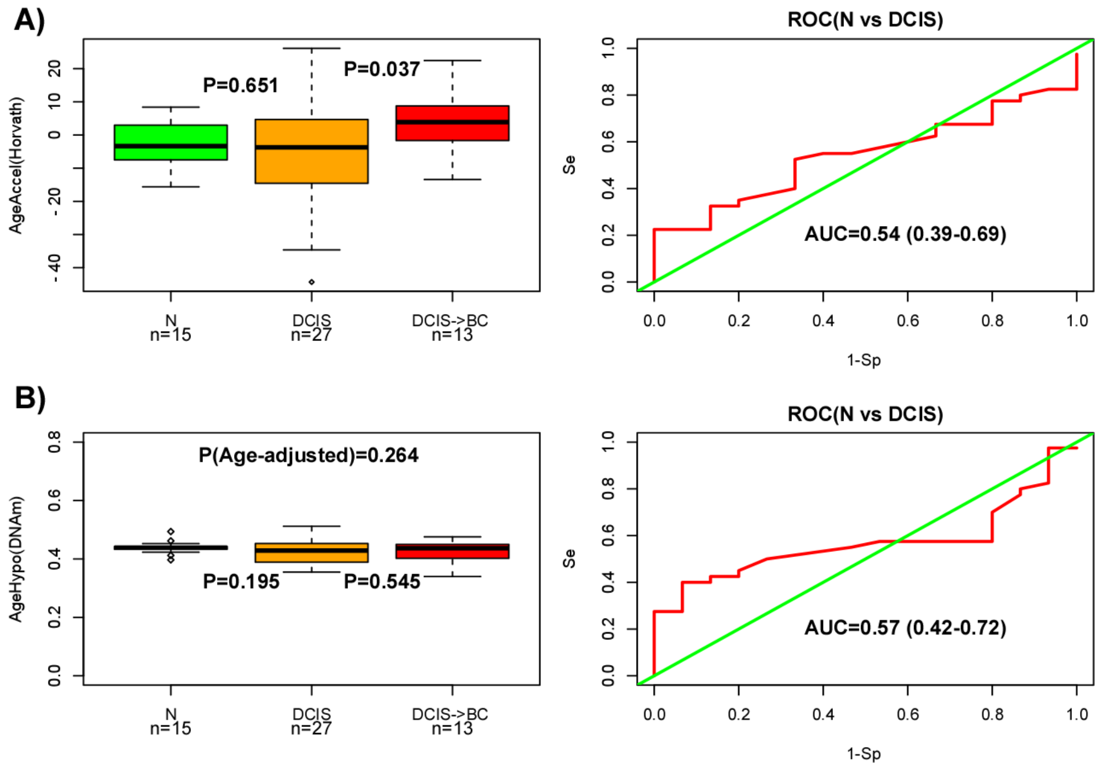

fig.S16. Horvath's clock and the age-hypomethylated based model do not predict age-acceleration in DCIS and can't discriminate normal from DCIS. A) Boxplot comparing Horvath's Age acceleration measure for normal breast tissue (N), ductal carcinoma in situ (DCIS) and DCIS samples that progressed to an invasive breast cancer (BC). Number of samples in each group given below boxplot. One-tailed Wilcoxon rank sum test between the neighboring groups are given. Right panel is the ROC for discriminating normal from all DCIS samples. AUC and 95%CI are given. **B)** Boxplot comparing HypoAge-score for normal breast tissue (N), ductal carcinoma in situ (DCIS) and DCIS samples that progressed to an invasive breast cancer (BC). Number of samples in each group given below boxplot. One-tailed Wilcoxon rank sum test between the neighboring groups are given, as well as the P-value from a linear regression of HypoAge vs. group, adjusted for chronological age. Right panel is the ROC for discriminating normal from all DCIS samples. AUC and 95%CI are given.

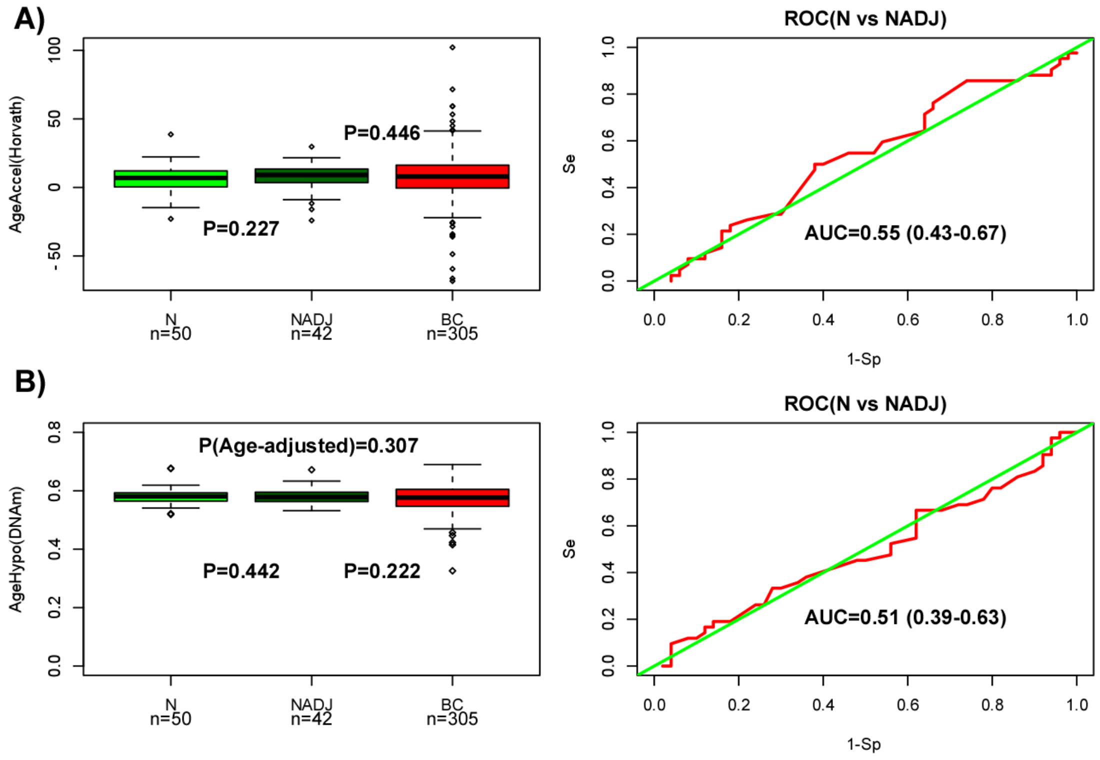

fig.S17. Horvath's clock and the age-hypomethylated based model do not predict age-acceleration in normal tissue adjacent to breast cancer and can't discriminate normal from normal-adjacent tissue. A) Boxplot comparing Horvath's Age acceleration measure for normal breast tissue (N), normal-adjacent tissue (NADJ) and breast cancer (BC) samples. Number of samples in each group given below boxplot. One-tailed Wilcoxon rank sum test between the neighboring groups are given. Right panel is the ROC for discriminating normal from normal-adjacent samples. AUC and 95%CI are given. **B)** As A), but now for HypoAge. One-tailed Wilcoxon rank sum test between the neighboring groups are given, as well as the P-value from a linear regression of HypoAge vs. group, adjusted for chronological age.

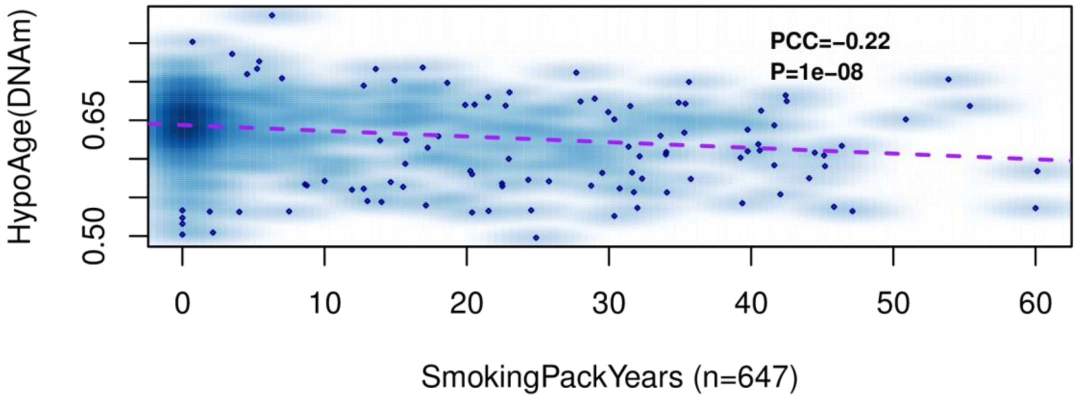

fig.S18. Density scatterplot of *HypoAge* (y-axis) vs smoking pack-years for a total of 647 buccal samples from women all aged 53 at sample draw. P-value is from a one tailed linear regression.

References

Ben-Porath, I., Thomson, M.W., Carey, V.J., Ge, R., Bell, G.W., Regev, A., and Weinberg, R.A. (2008). An embryonic stem cell-like gene expression signature in poorly differentiated aggressive human tumors. Nature genetics *40*, 499-507.

Hannum, G., Guinney, J., Zhao, L., Zhang, L., Hughes, G., Sadda, S., Klotzle, B., Bibikova, M., Fan, J.B., Gao, Y.*, et al.* (2013). Genome-wide methylation profiles reveal quantitative views of human aging rates. Molecular cell *49*, 359-367.

Liu, Y., Aryee, M.J., Padyukov, L., Fallin, M.D., Hesselberg, E., Runarsson, A., Reinius, L., Acevedo, N., Taub, M., Ronninger, M.*, et al.* (2013). Epigenome-wide association data implicate DNA methylation as an intermediary of genetic risk in rheumatoid arthritis. Nature biotechnology *31*, 142-147.

Reynolds, L.M., Taylor, J.R., Ding, J., Lohman, K., Johnson, C., Siscovick, D., Burke, G., Post, W., Shea, S., Jacobs, D.R., Jr.*, et al.* (2014). Age-related variations in the methylome associated with gene expression in human monocytes and T cells. Nature communications *5*, 5366.

Rhodes, D.R., Yu, J., Shanker, K., Deshpande, N., Varambally, R., Ghosh, D., Barrette, T., Pandey, A., and Chinnaiyan, A.M. (2004). Large-scale meta-analysis of cancer microarray data identifies common transcriptional profiles of neoplastic transformation and progression. Proceedings of the National Academy of Sciences of the United States of America *101*, 9309-9314.
